# Transgene-free genome editing in citrus and poplar trees using positive and negative selection markers

**DOI:** 10.1007/s00299-025-03627-2

**Published:** 2025-10-22

**Authors:** Dhiôvanna Corrêia Rocha, Miracle Osazee Omoregbee, Danyel Fernandes Contiliani, Rushil Mandlik, Gen Li, Juliet Mascoveto, Gary Coleman, James N. Culver, Daniel Rodriguez Leal, Alessandra Alves de Souza, Yiping Qi

**Affiliations:** 1 Department of Plant Science and Landscape Architecture, University of Maryland, College Park, MD 20742 USA; 2 Citrus Research Center “Sylvio Moreira”–Agronomic Institute (IAC), Cordeirópolis, SP Brazil; 3 Institute of Biology, State University of Campinas (UNICAMP), Campinas, SP Brazil; 4 Sugarcane Research Center–Agronomic Institute (IAC), Ribeirão Preto, SP Brazil; 5 Graduate Program in Genetics, Ribeirão Preto Medical School, University of São Paulo, Ribeirão Preto, SP Brazil; 6 Institute for Bioscience and Biotechnology Research, University of Maryland, Rockville, MD 20850 USA

**Keywords:** Cytosine base editing, Multiplexed co-editing, ALS, FCY-UPP, Citrus, Poplar, Transgene-free

## Abstract

**Key message:**

Transgene-free genome editing of the gene of interest in citrus and poplar has been achieved by co-editing the *ALS* gene via transient transgene expression of an efficient cytosine base editor.

**Abstract:**

CRISPR-Cas genome editing systems have been widely used in plants. However, such genome-edited plants are nearly always transgenic in the first generation when *Agrobacterium*-mediated transformation is used. Transgene-free genome-edited plants are valuable for genetic analysis and breeding as well as simplifying regulatory approval. It can be challenging to generate transgene-free genome-edited plants in vegetatively propagated or perennial plants. To advance transgene-free genome editing in citrus and poplar, we investigated a co-editing strategy using an efficient cytosine base editor (CBE) to edit the *ALS* gene to confer herbicide resistance combined with transient transgene expression and potential mobile RNA-based movement of CBE transcripts to neighboring, non-transgenic cells. An FCY-UPP based cytotoxin system was used to select non-transgenic plants that survive after culturing on 5-FC containing medium. While the editing efficiency is higher in poplar than in citrus, our results show that the CBE-based co-editing strategy works in both citrus and poplar, albeit with low efficiency for biallelic edits. Unexpectedly, the addition of the TLS mobile RNA sequence reduced genome editing efficiency in both transgenic and non-transgenic plants. Although a small fraction of escaping plants is detected in both positive and negative selection processes, our data demonstrate a promising approach for generating transgene-free base-edited plants.

**Supplementary Information:**

The online version contains supplementary material available at 10.1007/s00299-025-03627-2.

## Introduction

The development of strategies that enable the production of edited, transgene-free plants is desirable because it reduces regulatory restrictions and enhances consumer acceptance of genome-edited varieties, thereby allowing their subsequent market integration (Gu et al. 2021; Lokya et al. 2025; Menz et al. 2020; Prado et al. 2024; Tuncel et al. 2025). This is especially true for vegetatively propagated crops and perennial species such as citrus and poplar. For these crops, genetic segregation of transgenes is difficult due to their lengthy life cycles and breeding systems compared to annual or biannual plants (Lokya et al. 2025; Prado et al. 2024). In poplar, the challenge is further compounded by its dioecious breeding system, making controlled outcrossing of transgenes while achieving homozygous edits nearly impossible (Müller et al. 2020; Montalvão et al. 2022).

To obtain T_0_ CRISPR-Cas edited and transgene-free plants suitable for clonal propagation, several strategies can be employed. One approach is the direct delivery of ribonucleoproteins (RNPs) into protoplasts via PEG or by biolistics (Bertini et al. 2025; Blumberg et al. 2025; Mahmoud et al. 2022; Liu et al. 2021; Su et al. 2023; Yang et al. 2023a, b; Zhang et al. 2021). Other strategies promote the self-elimination of transgenes (Yang et al. 2025; Wu et al. 2025) or use plant virus-based vectors for transient expression of Cas9 and sgRNAs (Ellison et al. 2020; Liu et al. 2024a, b; Qiao et al. 2025). This often relies on the use of GFP as a marker to identify the presence of transgenes (Huang et al. 2022, 2023; Jia et al. 2024). However, these methodologies exhibit inherent limitations, such as the recalcitrance of many species and genotypes to regeneration from protoplasts, the absence of an in vitro selection system for edited shoots due to the absence of selection genes, and the substantial chimerism induced by techniques like biolistics (Awasthi et al. 2022; Bertini et al. 2025; Blumberg et al. 2025; Liu et al. 2021; Su et al. 2023; Huang et al. 2022, 2023; Jia et al. 2024; Yang et al. 2025; Wu et al. 2025).

The use of *Agrobacterium tumefaciens* to transfer genes of interest into plants is already a well-established practice across various crops, including citrus and poplar (Conti et al. 2021; Dominguez et al. 2022; Dutt et al. 2009; Fillatti et al. 1987; Li et al. 2023; Lu et al. 2025; Singh et al. 2023; Wen et al. 2022; Yevtushenko et al. 2010), representing the most convenient and widely used approach for delivering T-DNA containing genome editing reagents (Tuncel et al. 2025). Traditionally, gene expression in plants using *A. tumefaciens* occurs when transgenes are stably integrated into the genome, leading to the production of transgenic plants. However, *A. tumefaciens* can also be used for the transient expression of genes (Chen et al. 2018; Gong et al. 2021; Wu et al. 2025; Hoengenaert et al. 2025; Kelly et al. 2025; Krenek et al. 2015; Tuncel et al. 2025; Umemoto et al. 2023; Zhang et al. 2024). This provides a possible approach for transgene-free genome editing. To investigate this possibility, it is imperative that, instead of conventional selection for transgenic plants (e.g., via antibiotic resistance), approaches enabling the selection of transgene-free plants be applied.

However, without using a T-DNA derived selection marker, it is possible to obtain escape plants that are transgene-free but not edited. Thus, there is a need to align transgene-free strategies with methods that also allow for the selection of edited events while saving time and effort in molecular screening methods. In previous studies, a base editing strategy targeting the *ALS* gene was applied, conferring resistance to sulfonylurea and imidazolinone herbicides (Zhang et al. 2019; Alquezar et al. 2022; Danilo et al. 2019; Hoengenaert et al. 2025; Huang et al. 2022, 2023; Jia et al. 2024; Veillet et al. 2019). For citrus, in addition to editing *ALS*, other expression cassettes for the Cas12a nuclease and sgRNAs were also included with the aim of co-editing the canker susceptibility gene *CsLOB1*, as simultaneous cytosine base editing (CBE) of both genes was not achievable (Huang et al. 2023; Jia et al. 2024). Nevertheless, the use of these two editing systems resulted in a high number of herbicide selection escapes and low editing efficiency. In the case of poplar, only the CBE system was employed, and just 7% of the regenerated plants obtained through herbicide selection showed base editing in both the *ALS* and *CCoAOMT1* genes (Hoengenaert et al. 2025), while approximately 9% of the chlorsulfuron-resistant plants were edited at both the *ALS* and *CEN* target sites (Wu et al. 2025).

In this study, we aimed to integrate an efficient CBE system, capable of simultaneously generating two edited genes, with strategies designed to select edited, transgene-free plants in tissue culture. For this purpose, we utilized vectors containing a highly efficient cytosine base editor (CBE) based on hA3A-Y130 cytidine deaminase that conferred efficient base editing in rice, tomato, and poplar (Li et al. 2021; Randall et al. 2021; Ren et al. 2021). In addition, we also explored the strategy of simultaneous tagging CBE mRNA and sgRNAs with a mobile RNA motif of the tRNA-like structure 2 (TLS2) (Zhang et al. 2016), which was a mobile RNA motif previously used for grafting-based genome editing in *Arabidopsis* and *Brassica napus* (Yang et al. 2023a, b), and for increasing the efficiency of virus-induced editing in wheat (Qiao et al. 2025). Our hypothesis is that the use of mobile RNAs may help increase genome editing in neighboring, non-transgenic cells during tissue culture.

**Fig. 1:**
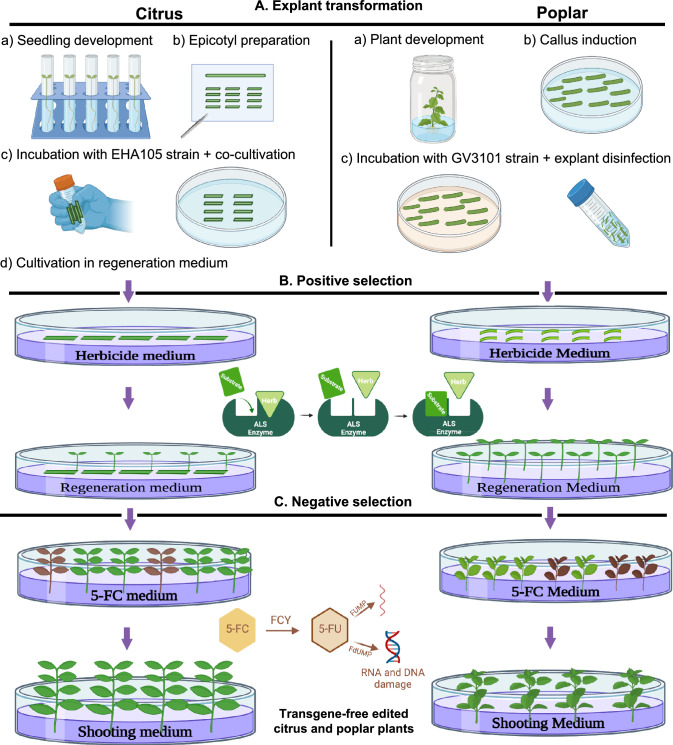
Transgene-free genome editing pipelines in citrus and poplar. **A** Genome editing is performed by transforming explants of citrus and poplar with *Agrobacterium tumefaciens*, which delivers the T-DNA carrying the co-editing reagents (simultaneous editing of *ALS* as the positive selection marker and a gene of interest) and the FUY-UPP toxin genes. **B** Positive selection via co-editing of the *ALS* gene, which confers chlorsulfuron resistance. In this strategy, a cytosine base editor (CBE) is used to introduce a codon change in the *ALS* coding sequence. This mutation prevents herbicide binding while preserving enzyme functionality. Consequently, only plantlets with successful editing are regenerated when cultured on an herbicide-containing medium. **C** Negative selection using the FCY-UPP toxic genes for transgene-free plants. The molecule 5-fluorouracil (5-FU) inhibits DNA synthesis by targeting the enzyme thymidylate synthase, which is essential for thymine biosynthesis. In the presence of the substrate 5-fluorocytosine (5-FC) and the enzyme cytosine deaminase (FCY), 5-FC is converted into 5-FU, which is cytotoxic. The cytotoxic effect is enhanced by co-expression of uracil phosphoribosyltransferase (UPP), which further converts 5-FU to 5-fluoroUMP. This system enables the negative selection of transgene-free plants: only those plants without transgene integration and continuous expression will survive in the presence of 5-FC. For citrus, epicotyls were incubated with *A. tumefaciens* strain EHA105 for 10 min, followed by a culture timeline consisting of co-cultivation (3 days), regeneration medium (1 week), positive selection (6 weeks), regeneration medium (8 weeks), negative selection (12 weeks), and shooting medium (4 weeks), totaling approximately 7 months. Cultures were maintained at 24 °C under a 16 h light (75 µmol m⁻^2^ s⁻^1^)/8 h dark photoperiod. For poplar, petiole explants from 8 to 10-day-old seedlings were transformed with *A. tumefaciens* strain GV3101 via overnight co-cultivation at 28 °C in the dark. The subsequent culture timeline included regeneration medium (2 weeks), positive selection (~ 6 months), and negative selection (11 days). Cultures were maintained at 23 °C under light 100 µmol m⁻^2^ s⁻.^1^

In our system for the generation of transgene-free genome edited plants (Fig. 1), the CBE was employed to edit the *ALS* gene, enabling their positive selection on media containing the herbicide chlorsulfuron. This CBE system was also used for the co-editing of the *CsNPR3* gene in citrus and the *Pt4CL1* gene in poplar. Introduction of a premature early stop codon predicted at the target site by the online tool CRISPR-BETS will help generate null alleles of both target genes (Wu et al. 2022). To test the utility of the selection of transgene-free plants, the *FLUOROCYTOSINE DEAMINASE* (*FCY*) and *URACIL PHOSPHORIBOSYL TRANSFERASE* (*UPP*) genes were incorporated into the vectors. In the presence of the 5-Fluorocytosine (5-FC) substrate, the enzymes encoded by these genes produce cytotoxic molecules, leading to cell death (Leonhardt et al. 2020; Stuttmann et al. 2021). Consequently, upon applying the substrate to the culture medium of regenerated plants, only those without T-DNA integration into their genome are capable of survival. This strategy enables the selection of transgene-free plants following the selection of the edited plants **(**Fig. 1**)**.

## Materials and methods

### Vectors construction

The mobile RNA TLS2 sequence was synthesized as a gene fragment by Integrated DNA Technologies (IDT) and then incorporated into the vectors pYPQ132B (Addgene #69282), pYPQ133B (Addgene #69283), pYPQ134B (Addgene #179216), and pYPQ265E2 (Addgene #164719, Ren et al. 2021) through a HiFi assembly reaction (NEBuilder DNA HiFi DNA Assembly Master Mix, New England Biolabs, Ipswich, MA, USA). Subsequently, the corresponding oligos for each sgRNA were designed, synthesized, annealed, and ligated into the vectors pYPQ132, pYPQ133, and pYPQ134 at BsmBI (Esp3I) sites using the T4 ligase enzyme. The expression cassette for the FCY-UPP was also synthesized and then inserted into the pYPQ131 vector (Addgene #69281) without the TLS2 sequence.

The expression T-DNA vectors were constructed by following the streamlined protocols based on Golden Gate and Gateway cloning (Lowder et al. 2015). To create a multiplexed sgRNA expression system along with the toxin genes, a Golden Gate reaction was performed with BsaI-HF and T4 ligase, producing the pYPQ144-FCY-UPP_ALS_NPR3_TLS2 for citrus and pYPQ143-FCY-UPP_ALS_4CL1_TLS2 for poplar. Finally, a Multisite Gateway LR reaction (with LR Clonase II from Invitrogen) was performed to form the T-DNA final vectors. For citrus, the entry vectors used in this reaction were pYPQ144-FCY-UPP_ALS_NPR3_TLS2 and pYPQ265E2-TLS2 made from pYPQ265-E2 (Addgene #164719), and the destination vector was pCGS710. For poplar, the entry vectors were pYPQ143-FCY-UPP_ALS_4CL1_TLS2 and pYPQ265E2-TLS2, and the destination vector was pYPQ202 (Addgene #86198; Tang et al. 2017). To assemble the vector without TLS2 sequences, the same procedures were followed, with the normal CBE and gRNA entry vectors devoid of TLS2. The products of these assemblies were the final vectors: pLR5432 without TLS and pLR5433 with TLS for citrus (Supplemental Fig. 1, Supplemental data 1 and 2); pLR5478 without TLS and pLR5479 with TLS for poplar (Supplemental Fig. 2, Supplemental data 3 and 4). All primers used for citrus and poplar are listed in Supplemental Tables 1 and 2, respectively.

### Citrus epicotyl transformation and positive selection

After assembling the vectors and propagating them in *Escherichia coli* (DH5α strain), *A. tumefaciens* EHA105 strain was used to carry out 10 genetic transformation experiments using citrus epicotyls, a protocol modified from a previous study (Caserta et al. 2014). Carrizo seeds (Lyn Citrus Seed, Arvin, CA) were cultured in vitro in the dark for four weeks to promote germination.

A transformed *Agrobacterium* (EHA105) colony carrying the binary vector of interest was streaked on solid LB media (supplemented with 50 mg/L kanamycin and 50 mg/L rifampicin) and incubated at 28 ºC for 3 days. The incubated colonies were scraped off the LB plates and grown overnight in 10 mL liquid LB media supplemented with 50 mg/L kanamycin and 50 mg/L rifampicin at 28 ºC. After reaching an OD600 of 0.4, *Agrobacterium* cells were harvested by centrifugation and resuspended in MS liquid medium (Murashige and Skoog 1962) to an adjusted OD600 of 1.

Epicotyl segments approximately 1 cm in length were cut at a bevel and incubated for 10 min with the *Agrobacterium*. The explants were then incubated for three days at 24 °C in the dark on MS medium supplemented with BAP (6-benzylaminopurine, 3 mg/L) and NAA (Naphthaleneacetic acid, 0.1 mg/L). After co-cultivation, the explants were transferred to petri dishes containing MS selection medium supplemented with BAP (3 mg/L), NAA (0.05 mg/L), and timentin (200 mg/L). Following a one-week period, positive selection of explants from four experiments (2 for each vector) was initiated by transferring them to MS medium supplemented with BAP (3 mg/L), NAA (0.05 mg/L), and the herbicide chlorsulfuron (40 µg/L). Citrus epicotyls were exposed to light one month after the transformation experiment. The temperature was maintained at 24 °C under a photoperiod of 16 h of white light and 8 h of darkness. The positive selection occurred for two rounds of three weeks each. For the other six experiments carried out (3 for each vector), positive selection started 55 days after the co-cultivation period and followed the same procedure.

### Poplar transformation and positive selection

In vitro plantlets of *Populus tremula* x *Populus alba* clone 717-1B4 plants were grown on LS media (Linsmaier and Skoog) containing 0.1 mg/L IBA at 23 ºC with continuous lighting at an intensity of approximately 100 µmol/m^2^/s. Poplar transformation was performed as described previously with modifications (Li et al. 2023). The petioles of one to two-month-old poplar plantlets are wounded and precultured on callus induction media (CIM) (DKW media supplemented with 1X MS vitamins, 1.5 mg/L NAA, 0.25 g/L BA, and 2.2 ug/L thidiazuron) for 8–10 days in the dark at 25 ºC. A transformed *Agrobacterium* (GV3101*)* colony carrying the binary vector of interest was streaked on solid LB media (supplemented with 50 mg/L kanamycin and 50 mg/L rifampicin) and incubated at 28 ºC for 2 days. The incubated colonies were scraped off the LB plates and grown overnight in 10 mL liquid LB media supplemented with 50 mg/L kanamycin, 50 mg/L rifampicin, and 5 μM acetosyringone at 28 ºC. After overnight incubation, *Agrobacterium* cells were harvested by centrifugation and resuspended in liquid callus induction media (CIM) to an OD_600_ of 0.3. 25 mL of resuspended and diluted *Agrobacterium* was added to a petri dish, and pre-cultured petiole explants were added to the petri dish and incubated overnight at 28 ºC in the dark with gentle agitation. After overnight co-cultivation of petiole explants with *Agrobacterium,* the explants were washed and blotted on sterile paper towels and then transferred to CIM. After two days of co-cultivation in the dark, the explants were disinfected by washing three times each with sterile water and CIM supplemented with 200 mg/L vancomycin, 250 mg/L cefotaxime, and 25 mg/L tetracycline. Disinfected explants were transferred to CIM supplemented with 250 mg/L cefotaxime and 200 mg/L timentin and incubated in the dark at room temperature for two weeks, after which they were then transferred to fresh CIM plates supplemented with 40 μg/L chlorsulfuron, 250 mg/L cefotaxime, and 200 mg/L timentin. After two weeks, explants were transferred to shoot induction media (SIM) (DKW supplemented with 1 × MS vitamins and 22.2 μg/L thidiazuron, 250 mg/L cefotaxime, 200 mg/L timentin, and 40 μg/L chlorsulfuron). Once regenerated shoots were of sufficient size, they were moved into root-induction media (RIM) (0.5X MS media, 15 g/L sucrose, 0.1 mg/L IBA, 250 mg/L cefotaxime, 200 mg/L timentin, and 40 μg/L chlorsulfuron).

### Transgene identification by PCR

Following positive selection and shoot development, the DNA of the citrus leaves was extracted using the Dellaporta method (Dellaporta et al. 1983). Subsequently, PCR reactions were performed using Q5^®^ High-Fidelity DNA Polymerase (New England Biolabs, Ipswich, MA, USA) to identify transgenic plants before applying the 5-FC substrate for negative selection of transgene-free plants. Initially, two primer pairs were used for all regenerated shoots: AtUBQ10-F + FCY-R, which anneals to the promoter of the toxin expression cassette and to the FCY sequence, and AtU3_NPR3-F + AttB2-R, which anneals to the sgRNA expression cassette (Supplementary Table 1).

For poplar, leaf tissues of fully regenerated rooted plantlets were used for DNA extraction following the CTAB method (Doyle et al. 1991). For transgene-free detection, a DNA fragment covering a segment of the zCas9 region of the T-DNA plasmid vector was amplified using the primers TFD-Pt5478zCas9-F1 + Pt5478zCas9-R1, and another fragment of the FCY-UPP region was amplified using primers TFD-Pt5478EUPP-F1 + TFD-Pt5478EUPP-R1 (Supplementary Table 2).

### Negative selection for transgene-free citrus and poplar

For negative selection of transgene-free citrus plants, shoots were cultured on MS medium supplemented with 5-fluorocytosine (5-FC). Four consecutive three-week selection cycles were performed using increasing concentrations of 5-FC: (1) 0.25 g/L, (2) 0.5 g/L, (3) 1.0 g/L, and (4) 1.0 g/L. In the case of poplar, rooted plantlets were transferred to root induction media (RIM) containing 0.25 g/L of 5-FC for 11 days.

### Genome editing characterization

Analysis of genome editing was mainly done by next-generation sequencing (NGS) of PCR amplicons, using the Illumina HiSeq2500 platform. The primer barcode system described by Liu et al. (2019), called Hi-TOM (High-throughput Tracking of Mutations), was used. For the first PCR round, primers specific to the target region of the genes were designed (Supplemental Tables 1 and 2). In the second PCR round, the Hi-TOM barcodes were added to the primers, used to identify each plant individually, allowing for bioinformatic separation after sequencing. All PCR reactions were conducted using the high-fidelity Q5 (New England Biolabs, Ipswich MA, USA). After the second round, the amplifications were checked by electrophoresis, and the samples were purified using the QIAQuick PCR (QIAGEN, Germantown MD, USA). The samples were sent for sequencing by the company Genewiz. For bioinformatic analysis, the CRISPRMatch software (You et al. 2018) was used to merge the FASTq files. Next, the CRISPRMatch Split tool was employed to separate the samples based on the barcodes, and finally, CRISPResso2 (Clement et al. 2019) was used to identify and quantify the mutations found according to the gene’s reference sequence. Sanger sequencing was further carried out to genotype select samples as necessary.

## Results

### Citrus positive selection with herbicide

**Fig. 2:**
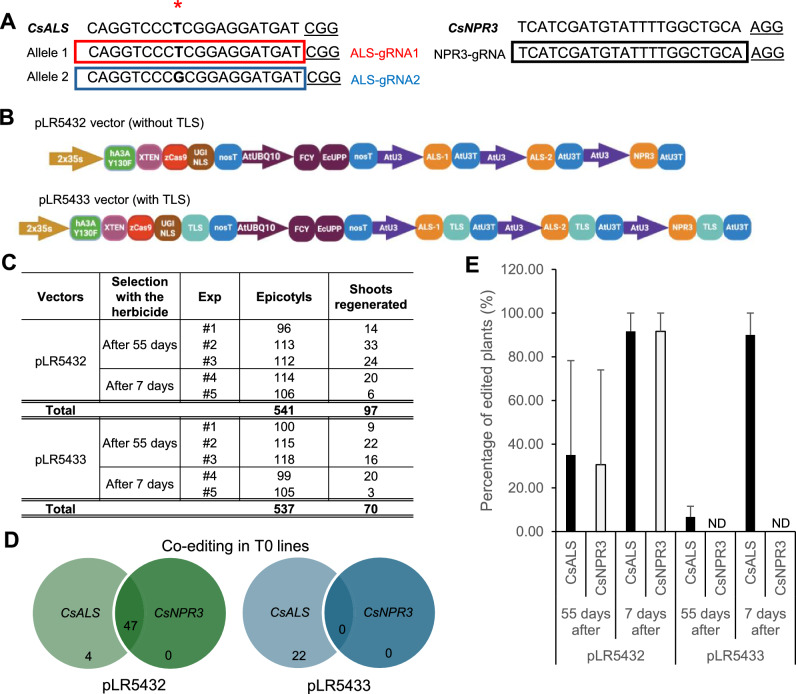
Positive selection of herbicide-resistant T0 citrus lines and co-editing analysis. **A** sgRNAs designed for *CsALS* and *CsNPR3* target sites. **B** Vector constructs for citrus transformation containing the base editing system, the FCY-UPP toxin genes, and the sgRNA expression cassettes. The pLR5432 vector differs from pLR5433 by the addition of TLS sequences to both CBE and sgRNAs. **C** Summary of transformation experiments performed for each vector and herbicide treatment. **D** A Venn diagram for the number of T0 edited citrus plants per gene and vector. **E** Percentage of edited plants obtained for each construct, separated by different herbicide treatment groups. “55 days after” refers to the results obtained when the herbicide treatment was started 55 days after cocultivation. “7 days after” refers to the results obtained when the herbicide treatment was started 7 days after cocultivation. ND indicates that the results were not detected. Error bars represent standard deviations

To edit the *CsALS* gene (ID: Cs_ont_7g012340, *C. sinensis* v3.0) and allow selection of edited plants in tissue culture, Sanger sequencing was first performed to confirm the target region. As previously demonstrated, Carrizo citrange carries two alleles of *CsALS* (Huang et al. 2022; Jia et al. 2024), which originate from its parental species, *Citrus sinensis* and *Poncirus trifoliata*. Based on the sequencing results (Supplemental Fig. 3A), two sgRNAs were designed to target each of the two alleles (Fig. 2A, Supplemental Table 3). To demonstrate co-editing of a gene of interest, a sgRNA was designed to target the *CsNPR3* gene (ID: Cs_ont_2g009270, *C. sinensis* v3.0) **(**Fig. 2A, Supplemental Table 3) (Tiwari et al. 2024), with the goal of introducing an early stop codon by the CBE to achieve gene knockout. Two multiplexed CBE vectors were generated, without and with the TLS2 mobile RNA motif because the mobile RNA may promote genome editing in surrounding non-transgenic cells throughout tissue culture (Fig. 2B). Following the explant transformation experiment, positive selection based on chlorsulfuron resistance was initiated 55 days post co-cultivation in the first three experiments. However, at this stage, small necrotic areas were observed, which could impair the development of edited shoots (Supplemental Fig. 3B). Therefore, in the two subsequent experiments, the chlorsulfuron selection was shifted to the beginning of the regeneration phase, seven days after co-cultivation (Supplementary Fig. 3B).

From the five experiments, a total of 97 plants were regenerated from the vector without TLS2 (pLR5432) and 70 plants from the vector with the TLS2 motif (pLR5433) (Fig. 2C). Since almost equal numbers of epicotyls were used for both vectors, it appears that pLR5432 (without the TLS2 motif) produced more herbicide-resistant plants than pLR5433 (with the TLS2 motif). For the experiments conducted with the pLR5432 vector, 51 plants showed editing in the *CsALS* gene, and 47 of these 51 plants also showed co-editing in the *CsNPR3* gene (Fig. 2D). No plants displayed sole editing of *CsNPR3* (Fig. 2D). These data suggest high efficiency of co-editing with the pLR5432 vector. The average percentage of edited plants, calculated based on the number of regenerated shoots, was 57.68 and 55.07% for each gene, respectively (Supplementary Fig. 3C). However, for the pLR5433 vector, only 22 plants (~ 30%) showed genome editing in the *CsALS* gene, and no plants showed editing in *CsNPR3* (Fig. 2D). Therefore, when comparing the number of edited plants generated by each vector, it appears that the use of TLS2 mobile RNA may compromise gene-editing efficiency in citrus.

Upon examining the plants obtained for each herbicide treatment, it became evident that the treatment initiated seven days post co-cultivation resulted in the highest number of edited plants (Fig. 2E). In this group, over 90% of the regenerated plants showed edits in the *CsALS* gene, indicating a low escape rate under this selection condition (Supplementary Fig. 3D). Hence, early treatment with herbicide is essential to prevent escapes.

### Citrus negative selection and genome editing characterization

**Fig. 3:**
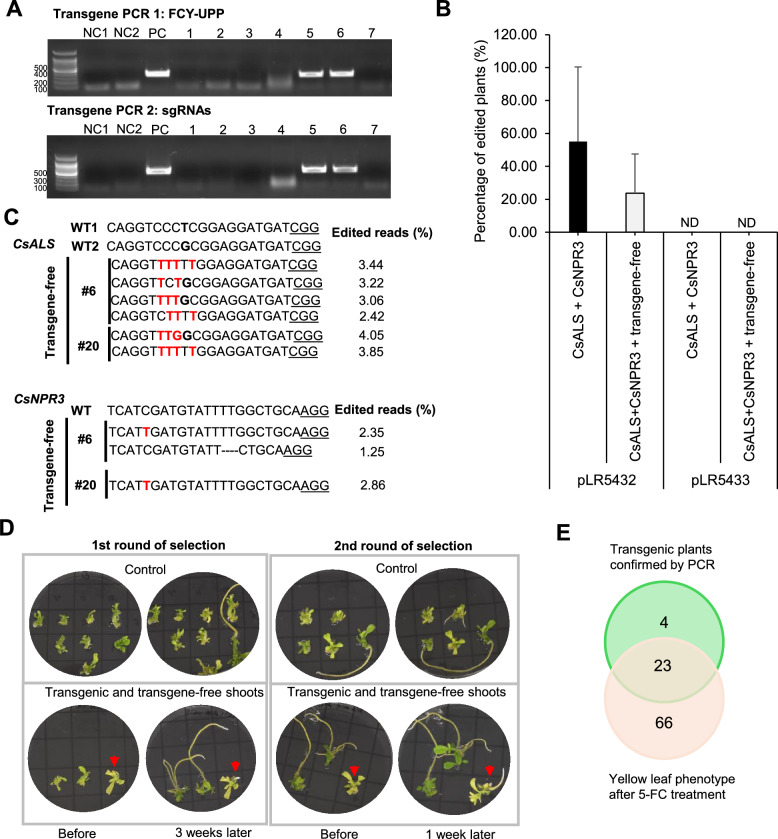
Analysis and selection of transgene-free genome editing events in T0 citrus plants. **A** Representative data for the confirmation of transgene-free events via PCR amplification of FCY-UPP fragment and a section of the sgRNA region of the T-DNA. *NC1* negative control using water. *NC2* negative control using WT shoot DNA. *PC* positive control using the DNA vector. 1–7: possible transformed shoots. **B** Percentage of plants edited at both target genes and free of T-DNA integration. ND indicates that the results were not detected. Error bars represent standard deviations. **C** Examples of transgene-free lines edited at both target genes and their corresponding editing efficiency based on the number of edited reads. **D** Results of negative selection after the second round of 5-FC application. Red arrows indicate PCR-positive shoots. **E** A Venn diagram showing the results of negative selection after the fourth round of 5-FC application. Green indicates the number of transgenic plants detected by PCR; the middle group represents PCR-positive plants that were affected by 5-FC treatment; and pink corresponds to plants that were affected by negative selection but were not PCR-positive

Before applying the 5-FC substrate to select transgene-free citrus plants, PCR analysis targeting two different regions of the T-DNA from the vectors was performed to identify transgenic plants (Fig. 3A). A total of 72 and 68 T-DNA-free plants were recovered from transformations with vectors pLR5432 and pLR5433, respectively. Among these, 24 plants transformed with the TLS-deficient vector (pLR5432) exhibited successful base editing at both target genes, representing 23.75% of the regenerated plants (Fig. 3B). In all edited plants, the intended C-to-T modification in the *CsALS* gene was achieved, leading to the substitution of the proline residue in the ‘QVPRRMI’ sequence with phenylalanine, which confers herbicide resistance. Likewise, the desired editing to introduce a stop codon in the *CsNPR3* gene was also successfully accomplished (Fig. 3C). Plants exhibiting between 0 and 30% edited reads are considered chimeric, those with > 30–70% edited reads are classified as monoallelic, and those with over 70% are considered biallelic. Based on this criterion that we established previously (Byiringiro et al. 2024), the results for the pLR5432 vector and frequency of editing of the *CsALS* gene indicate that the majority of the regenerated plants were likely chimeric, which is common in citrus species (Caserta et al. 2017; Jia et al. 2024; Sakar et al. 2024). As a result, 9 plants were classified as monoallelic, and 2 as biallelic (Table 1), while the rest were chimeric (Fig. 3C and Table 1). All monoallelic and biallelic plants were positive in PCR-based transgene detection, suggesting that the insertion of T-DNA into the plant genome and subsequent expression enhances genome editing efficiency. For the pLR5433 vector, the same pattern was observed: the majority of plants edited for the *CsALS* gene were chimeric, all of which were transgene-free, while the only monoallelic plant was transgenic (Table 1). Lastly, all plants edited for the *CsNPR3* gene exhibited a chimeric pattern, with most being transgene-free. These results showed that the positive selection strategy worked efficiently for the selection of co-edited plants when the standard vector was used.

**Table 1:** Genotype analysis of citrus lines based on the percentage of edited reads from NGS data

		*CsALS*			*CsNPR3*		
Vectors	Genotyping and editing (%)	Number of edited plants	Transgenic	Transgene-free	Number of edited plants	Transgenic	Transgene-free
pLR5432 (without TLS)	Chimeric (0–30%)^a^	40	26 (26.80%)^b^	14 (14.43%)	47	22 (22.68%)	25 (25.77%)
	Monoallelic (> 30–70%)	9	8 (8.24%)	1 (1.03%)	0	0 (0%)	0 (0%)
	Biallelic (> 70%)	2	2 (2.06%)	0 (0%)	0	0 (0%)	0 (0%)
pLR5433 (with TLS)	Chimeric (0–30%)	21	0 (0%)	21 (30%)	0	0 (0%)	0 (0%)
	Monoallelic (> 30–70%)	1	1 (1.43%)	0 (0%)	0	0 (0%)	0 (0%)
	Biallelic (> 70%)	0	0 (0%)	0 (0%)	0	0 (0%)	0 (0%)

^a^Percentage of edited reads per plant

^b^Percentage of edited plants calculated based on the number of regenerated shoots

To test negative selection in citrus, the 5-FC substrate was used with the goal of selecting only transgene-free plants. An initial concentration of 0.25 g/L of 5-FC was added to MS culture medium, and the plants were incubated for 3 weeks. Following 5-FC treatment, besides yellowing of leaves, no significant plant damage or death was observed (Fig. 3D). To enhance selection stringency, the concentration of 5-FC substrate was doubled to 0.5 g/L, and after one week, it was observed that transgenic plants exhibited further reduced growth and enhanced leaf yellowing (Fig. 3D). Consequently, two additional selection cycles with 1 g/L 5-FC were performed. Following selection with increased levels of 5-FC, 23 PCR-confirmed transgenic plants developed yellowing, 4 plants escaped the negative selection, and 66 PCR-negative plants also exhibited yellowing (Fig. 3E). Under this concentration, even control plants occasionally exhibited stress symptoms (Supplementary Fig. 4), suggesting off-target cytotoxicity. Therefore, the use of 5-FC as a negative selection marker did not appear to be robust compared to the PCR-based screen of transgene-free plants.

### Poplar positive selection with herbicide

**Fig. 4:**
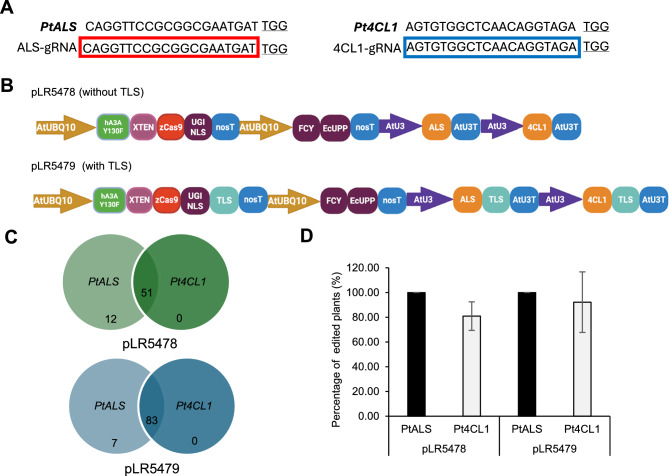
Positive selection of herbicide-resistant T0 poplar lines and co-editing analysis. **A** sgRNAs designed for *PtALS* and *Pt4CL1* target sites. **B** Vector constructs for poplar transformation, containing the base editing system, the FCY-UPP toxin genes, and the sgRNA expression cassettes. The pLR5478 vector differs from pLR5479 only by the addition of TLS sequences to both CBE and sgRNAs. **C** Venn diagrams showing the number of T0 plants exhibiting edits at both *PtALS* and *Pt4CL1* target sites for each construct. **D** Percentage of plants edited at each target site per vector construct. Error bars represent standard deviations

For poplar, the co-editing strategy targeted both *Pt4CL1* (ID: PtXaTreH.01G031400x PtXaAlbH.01G031600) and *PtALS* (ID: PtXaTreH.15G077800xPtXaAlbH.15G077900). The *Pt4CL1* gene encodes 4-coumarate:coenzyme A ligase, which is a key enzyme in the phenylpropanoid pathway and lignin biosynthesis (Hu et al. 1999). We have successfully used CBE to edit *Pt4CL1* and demonstrated this approach as a more environmentally sustainable strategy to generate super strong engineered wood (Li et al. 2021; Liu et al. 2024a, b). The same *Pt4CL1* sgRNA used in these previous studies was also used here. The sgRNAs for *PtALS* and *Pt4CL1* target sites (Fig. 4A, Supplemental Table 3) were multiplexed into two vector constructs. As in citrus, we tested two strategies, one without the mobile RNA TLS2 (vector pLR5478) and one with TLS2 (pLR5479) (Fig. 4B). Two sets of transformations were performed using poplar petiole explants for each vector construct (pLR5478 or pLR5479), and edited shoots were regenerated in the presence of chlorsulfuron, resulting in 63 shoots regenerated for the vector without the TLS2 motif (pLR5478) and 90 shoots regenerated for the vector with the TLS2 motif (pLR5479) (Fig. 4C).

All plants regenerated after positive selection were edited at the *PtALS* target site for both constructs, meaning that poplar did not have any escape from the herbicide selection. However, at the *Pt4CL1* target site, 51 out of 63 T_0_ plants showed editing from the vector without TLS2, while 83 out of 90 T0 plants showed editing from the vector with TLS2 (Fig. 4C). This means that the percentages of co-edited plants are very high, at 80.95% for pLR5478 and 92.22% for pLR5479 (Fig. 4D).

### Poplar negative selection and genome editing characterization

**Fig. 5:**
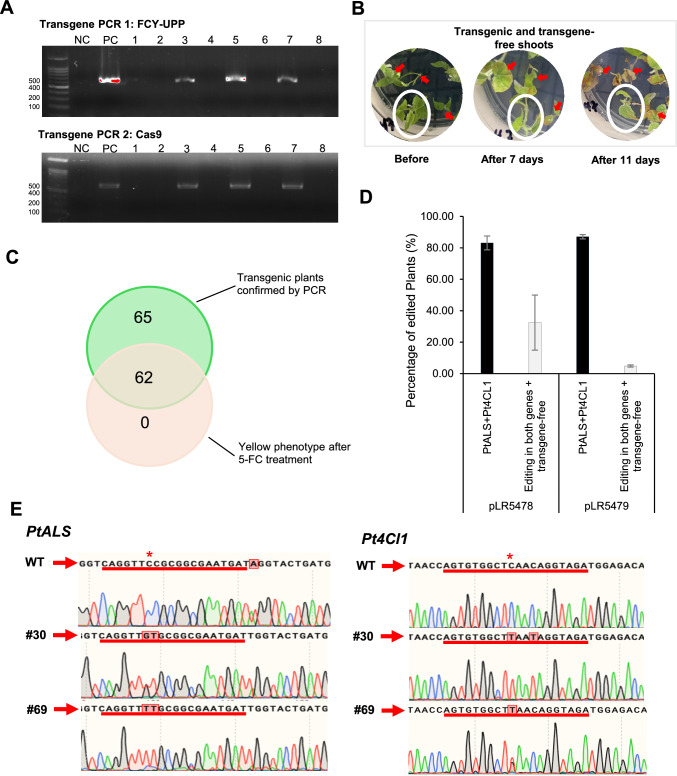
Analysis of transgene-free genome editing in T0 poplar plants and negative selection. **A** Representative data for the confirmation of transgene-free events via PCR amplification of FCY-UPP fragment and a section of the zCas9 region of the T-DNA. NC: negative control using water. PC: positive control using the DNA vector. 1–8: possible transformed shoots. **B** Negative selection of transgenic-free plants with 0.25 g/L of 5-FC substrate. The red arrows indicate transgenic plants detect by PCR, and the plant in the white circle indicates the transgene-free plant. Pictures of the plants taken 7 and 11 days after 5-FC treatment. **C** A Venn diagram showing the evaluation of two selection methods for transgene-free plants. Green circle indicates the number of transgenic plants detected by PCR; the middle group represents PCR-positive plants that were affected by 5-FC treatment; and pink circle corresponds to plants that were affected by negative selection but were not PCR-positive. **D** Percentage of plants edited at both target genes and free of T-DNA integration. Error bars represent standard deviations. **E** Examples of Sanger sequencing chromatograms of transgene-free plant samples showing *PtALS* and *Pt4CL1* modifications

To assess transgene integration, PCR was performed to amplify two regions of the T-DNA, and it showed that 127 out of 153 plantlets were transgenic (Fig. 5A). Additionally, the negative selection strategy using 5-fluorocytosine (5-FC) was also used to select against T-DNA integration. This was accomplished by transferring plants from the positive selection culture media containing chlorsulfuron to the negative selection media containing 0.25 g/L 5-FC (Hoengenaert et al. 2025). Following transfer to 5-FC containing media, leaf necrosis was observed 7 days after transfer (Fig. 5B). Consistent with the presence of T-DNA, 62 of the PCR-positive plants began to die, yet 65 PCR-positive shoots showed no effect to 5-FC treatment (Fig. 5C). However, after 14 days, all the plants began to exhibit signs of necrosis, including the transgene-free plants. This suggests that poplar is likely to be more sensitive to 5-FC treatment compared to citrus. We suspect that the differences in response to 5-FC might be related to expression levels of the FCY and EcUPP transgenes, where plants that were initially killed by 5-FC might have high expression of these transgenes. Hence, the 5-FC treatment is limited in its ability to select transgene-free plants in poplar.

Of 63 regenerated shoots transformed with the pLR5478 vector lacking the TLS2 mobile RNA motif, 15 were confirmed to be both transgene-free and successfully edited at the two target sites, representing a genome co-editing efficiency of 32.5% (Fig. 5D). In contrast, only 4 of 90 (4.86% efficiency) regenerated shoots transformed with the TLS2 vector with the mobile RNA motif were found to be transgene-free and co-edited (Fig. 5C). These results indicate that the mobile RNA motif does not appear to increase the efficiency of recovering transgene-free genome-edited plantlets and may actually reduce efficiency.

In terms of zygosity, of the transgenic plants transformed with the vector lacking the mobile RNA motif (pLR5478), 35 and 30 shoots were monoallelic or biallelic for edits (including homozygous edits) at the *PtALS* and *Pt4CL1* sites, respectively, while 6 shoots showed chimerism for each gene (Table 2). Among transgene-free plants, 13 and 2 shoots displayed monoallelic or biallelic edits, and 9 and 13 were chimeric for *PtALS* and *Pt4CL1*, respectively (Table 2). For the TLS2-motif containing vector, 62 and 52 transgenic plants exhibited monoallelic or biallelic edits at the respective loci, while 24 and 27 were chimeric for the *PtALS and Pt4CL1* target sites, respectively (Table 2). In the transgene-free group, 3 and 4 shoots showed monoallelic or biallelic patterns for *PtALS* and *Pt4CL1*, respectively, and only one plant exhibited a chimeric pattern for *PtALS* (Table 2).

**Table 2:** Genotype analysis of poplar lines based on the percentage of edited reads from NGS data

		*PtALS*			*Pt4CL1*		
Vectors	Genotype and editing (%)	Number of edited plants	Transgenic	Transgene-free	Number of edited plants	Transgenic	Transgene-free
pLR5478 (without TLS)	Chimeric (1–30%)^a^	15	6 (9.52%)^b^	9 (14.29%)	19	6 (11.76%)	13 (25.49%)
	Monoallelic (> 30–70%)	7	3 (4.76%)	4 (6.35%)	9	8 (15.69%)	1 (1.96%)
	Biallelic (> 70%)	41	32 (50.79%)	9 (14.29%)	23	22 (43.14%)	1 (1.96%)
pLR5479 (with TLS)	Chimeric (1–30%)	25	24 (26.67%)	1 (1.11%)	27	27 (32.53%)	0 (0%)
	Monoallelic (> 30–70%)	16	14 (15.56%)	2 (2.22%)	13	9 (10.84%)	4 (4.82%)
	Biallelic (> 70%)	49	48 (53.33%)	1 (1.11%)	43	43 (51.81%)	0 (0%)

^a^Percentage of edited reads per plant

^b^Percentage of edited plants calculated based on the number of regenerated shoots

Sanger sequencing was used to further confirm the editing in a transgene-free line from each vector and included line 30 derived from pLR5478 and line 69 derived from pLR5479. These two lines were selected based on NGS data, which indicated that line 30 was biallelic at the *Pt4CL1* target site with an efficiency of 89.67%, while the editing at the *PtALS* site was 31%. NGS sequencing of line 69, transformed with the TLS2-tagged vector, showed monoallelic editing at both target sites, with an efficiency of 40.9% at the *Pt4CL1* target site and 69.97% at the *PtALS* target site. The editing results were indeed confirmed by Sanger sequencing (Fig. 5E). Subsequently, we identified line 30 as a transgene-free homozygous line for the *Pt4CL1* edit, suggesting that our co-editing strategy successfully generated a loss-of-function mutant for the target gene of interest (*4CL1*) within one generation.

## Discussion

In this research, we developed a methodology to obtain edited and transgene-free citrus and *Populus* (poplar) trees in the T_0_ generation. This approach is notable for its simplicity and effectiveness, relying on transient expression of *Agrobacterium*-delivered vectors and selective regeneration of edited shoots in tissue culture. It is particularly advantageous for vegetatively propagated perennial species, as the technology can be used to rapidly develop commercially valuable traits. A co-editing strategy was employed (Veillet et al. 2019), enabling simultaneous modification of two genes at the same time. One gene, *ALS*, acts as a selectable marker when edited, which allows for selection of edited plants by resistance to the herbicide chlorsulfuron. This is combined with editing of a second gene of interest, which in this research included *CsNPR3* for citrus and *Pta4CL1 for* poplar. Similar co-editing systems reported for citrus resulted in a low number of edited plants and a high number of herbicide escape plants (Huang et al. 2023; Jia et al. 2024). In these previous reports, only four transgene-free citrus plants edited in both *CsALS* and *CsLOB1* genes were obtained, resulting in efficiencies equivalent to 1.9 and 5.2% in each respective study (Supplemental Table 4). In contrast, our study generated 24 transgene-free citrus plants edited in both targets, achieving a co-editing efficiency of 23.75%, representing a 4.5-fold increase compared to similar previous reports (Supplemental Table 4). However, we did not obtain transgene-free citrus plants with biallelic editing in both target genes. When compared with the work of Su et al. (2023), in which RNP transfection into protoplasts resulted in 38 plants with biallelic editing (97.4% efficiency), it is noted that the protoplast-based method for citrus is more efficient. However, although regeneration protocols for citrus protoplasts do exist (Grosser and Gmitter 1990; Omar et al. 2016; Soriano et al. 2022), the process is lengthy, complex, and highly prone to contamination due to the nutrient-rich media required. Often, the high efficiency RNP-based genome editing in citrus protoplasts could not be easily transferred into whole plants due to failed regeneration (Fang et al. 2023).

A similar increase in editing efficiency and transgene-free plant recovery was also observed in poplar by recent reports: Hoengenaert et al. (2025) reported that 7% of regenerated plants were T-DNA-free with co-editing at the two targets (*PtALS* and *CCoAOMT1*), while Wu et al. (2025) reported 26.7% transgene-free plants that were edited at both target sites (*PdbALS* and *CEN*) with only ~ 7% of chlorsulfuron-resistant lines having homozygous edits at the *CEN* locus. In contrast, our approach yielded 32.45% of regenerated poplars that were co-edited and transgene-free, including ~ 2% that were biallelic (Supplemental Table 5).

This improved performance in co-editing could be partly attributed to the use of the hA3A-Y130F base editing system, which was shown to be highly efficient in rice, *Arabidopsis*, tomato, and poplar compared to other deaminases, such as rAPOBEC1, which was previously used for citrus (Li et al. 2021; Randall et al. 2021; Ren et al. 2021; Wang et al. 2018). It would be worth testing other high-efficiency CBEs in the future (Contiliani et al. 2025; Fan et al. 2024; Huang et al. 2024; Liu et al. 2025). In addition, the vector used in this study includes the AtU3 promoter to drive sgRNA expression, whereas the use of a defective AtU6 promoter may lead to low-efficiency genome, as demonstrated before (Deguchi et al. 2025; Li et al. 2021; Randall et al. 2021). In this regard, strategies for enhancing sgRNA expression may be explored to improve the overall CBE-based co-editing efficiency.

It is necessary to further improve the editing efficiency in our systems. We observed a high frequency of chimeric editing resulting in plants composed of a mixture of edited and non-edited cells (Frank and Chitwood 2016; Song et al. 2022), rather than uniformly edited tissues with monoallelic or biallelic editing. In annual or biennial species propagated by seeds, individuals with homozygous or biallelic modifications can be selected through successive generations (Cardi et al. 2023; Prado et al. 2024). However, this strategy is not feasible for vegetatively propagated plants. Therefore, strategies aimed at achieving higher editing efficiency while reducing chimerism are highly beneficial for producing non-chimeric plants. To enhance editing efficiency, highly efficient base editors can be applied. In a recent study, Contiliani et al. (2025) examined various cytidine deaminases from different species and discovered that the Orca-derived OoA3GX2 enzyme demonstrated higher base-editing efficiency than commonly used CBEs, highlighting the vast range of cytidine base editors that can be tested in woody species. Also, using two UGIs instead of one was demonstrated to improve base editing precision in plants (Ren et al. 2021; Yu et al. 2020). It is equally appealing to explore strategies to bolster the expression of CRISPR-Cas9 CBE systems. For example, an intronized Cas9 may be used to enhance the CBE expression (Grützner et al. 2020; Villette et al. 2024). Strong Pol II promoters, rather than Pol III promoters, may be used to drive sgRNA expression, either in a single transcript unit or coupled with ribozyme processing or tRNA processing (Gao et al. 2014; Tang et al. 2019; Xie et al. 2015).

In this study, we also attempted transgene-free genome editing with the mobile TLS sequence. Our hypothesis was that this mobile RNA will help move the mRNA of CBE and sgRNAs to neighboring cells that lack T-DNA integration. Mobile RNAs are known for their ability to move from cell to cell, via plasmodesmata, or to various tissues of the plant through phloem transport, and even between plants and fungal parasites (Park et al. 2021; Zhang et al. 2016). Moreover, Yang et al. (2023a, b) demonstrated the use of TLS for generating transgene-free edited plants via grafting. In that study, two types of TLS sequences (TLS1 and TLS2) were tested, with TLS2 yielding a greater number of edited scion plants. Consequently, TLS 2 (tRNAMet-ΔDT), characterized by the absence of D and T loops, was utilized in our work. However, our results using citrus and poplar consistently showed that the addition of this TLS to our CBE system reduced its editing efficiency. Hence, it is unclear whether any of the TLS-tagged CBE mRNA and sgRNAs moved from cell to cell to promote editing in adjacent cells as we hypothesized. Our results suggest that the use of TLS sequences to promote editing in adjacent cells may be at the cost of RNA stability and editing efficiency in general, which may explain the extremely low editing efficiency reported for TLS-based grafting-mediated genome editing (Yang et al. 2023a, b). However, this warrants more investigation with additional CRISPR-Cas based editing systems and in more plant species.

Another major aspect of our study is the development of a counter, negative selection system for transgene-free plants. We adopted an FCY-UPP system (Anderson et al. 1989; Tiraby et al. 1998), in which the *FCY* gene is from *Saccharomyces cerevisiae* and the *UPP* gene is from *E. coli*. The combined use of the *ScFCY* and *UPP* system for inducing cell death in plants was first demonstrated by Leonhardt et al. (2020) in *Arabidopsis thaliana*. However, in the context of genome editing, the FCY-UPP was first tested by Stuttmann et al. (2021), in which the system enabled the identification of three transgene-free *Nicotiana benthamiana* seeds from a pool of 150 T1 generation seeds. We found that 5-FC concentration of 0.25 g/L had no observable effect on citrus shoots. At 0.5 g/L, the substrate began to affect transgenic plants, while at 1 g/L, even control (non-transgenic) shoots were impacted. At this higher concentration, 23 out of 27 PCR-confirmed transgenic plants were visibly affected by the substrate. These results also suggest the possibility of optimizing 5-FC concentration, likely between 0.5 and 1 g/L, for more effective negative selection of citrus transgene-free plants. Our study represents the first application of the FCY-UPP system in citrus.

In poplar, a similar system had recently been used by Hoengenaert et al. (2025), but without demonstrated success in screening regenerated plants. In that study, only the *CodA* gene from *E. coli,* which is functionally analogous to *ScFCY* used in this study, was employed. However, the authors focused solely on identifying a concentration capable of inducing or inhibiting callus regeneration from transgenic and non-transgenic explants and did not examine the effect on plant regeneration. In contrast, in our study, we applied the 5-FC substrate directly to regenerated plantlets after positive selection as opposed to during callus induction. As a result, approximately half of the transgenic poplar plants exhibited visible symptoms following 5-FC application, suggesting that applying the substrate in this way may be more effective for counter-selection. Compared to CodA, our FCY-UPP system includes UPP, which encodes an uracil phosphoribosyl transferase that further converts 5-FU to 5-fluoroUMP. As expected, the combination of FCY and UPP in our system appeared to increase sensitivity to 5-FC, likely due to the rapid accumulation of toxic 5-fluoroUMP within the cells. However, based on our data, it is still necessary to reduce the number of escaped plants to improve the negative selection efficiency. This may be done by optimizing the concentration of 5-FC applied and the timing of the application in the procedure (Fig. 1). With that, we hope more reliable protocols of using FCY-UPP based negative selection will work robustly for the selection of transgene-free genome edited citrus and poplar plants.

In conclusion, we assessed *Agrobacterium*-mediated co-editing strategies for transgene-free editing in citrus and poplar with a combination of positive and negative selection markers. The approach without the use of TLS mobile sequences led to a high number of transgene-free edited poplar and citrus plants, with simultaneous edits in *ALS* and the gene of interest. Editing efficiency was higher in poplar than in citrus, suggesting species-dependent outcomes. Nonetheless, this is the first study demonstrating the feasibility of solely using base editing for co-editing in citrus.

## Supplementary Information

Below is the link to the electronic supplementary material.

Supplementary file1 Supplementary information is available online.

Supplementary file2 (DOCX 60 kb)

## Data Availability

The modular co-editing vectors for citrus and poplar are made available at Addgene: pYPQ133B-TLS (#245882), pYPQ132B-TLS-PtALSgRNA (#245881), pYPQ132B-PtALSgRNA (#245880), pYPQ134B-TLS (#245879), pYPQ133B-TLS-CsALSgR1.2 (#245878), pYPQ132B-TLS-CsALSgR1.1 (#245877), pYPQ133B-CsALSgR1.2 (#245876), pYPQ132B-CsALSgR1.1 (#245875), and pYPQ131-FCY-UPP (#245873).
