# Supplementary material for: Transgene-free genome editing in citrus and poplar trees using positive and negative selection markers
Source: Plant Cell Rep. 2025 Oct 22;44(11):244. doi: 10.1007/s00299-025-03627-2 (PMC12546424; doi:10.1007/s00299-025-03627-2)
Supplement: Supplementary file 1 — Supplementary file1 Supplementary information is available online. [file 299_2025_3627_MOESM1_ESM.pptx]

## Slide 1
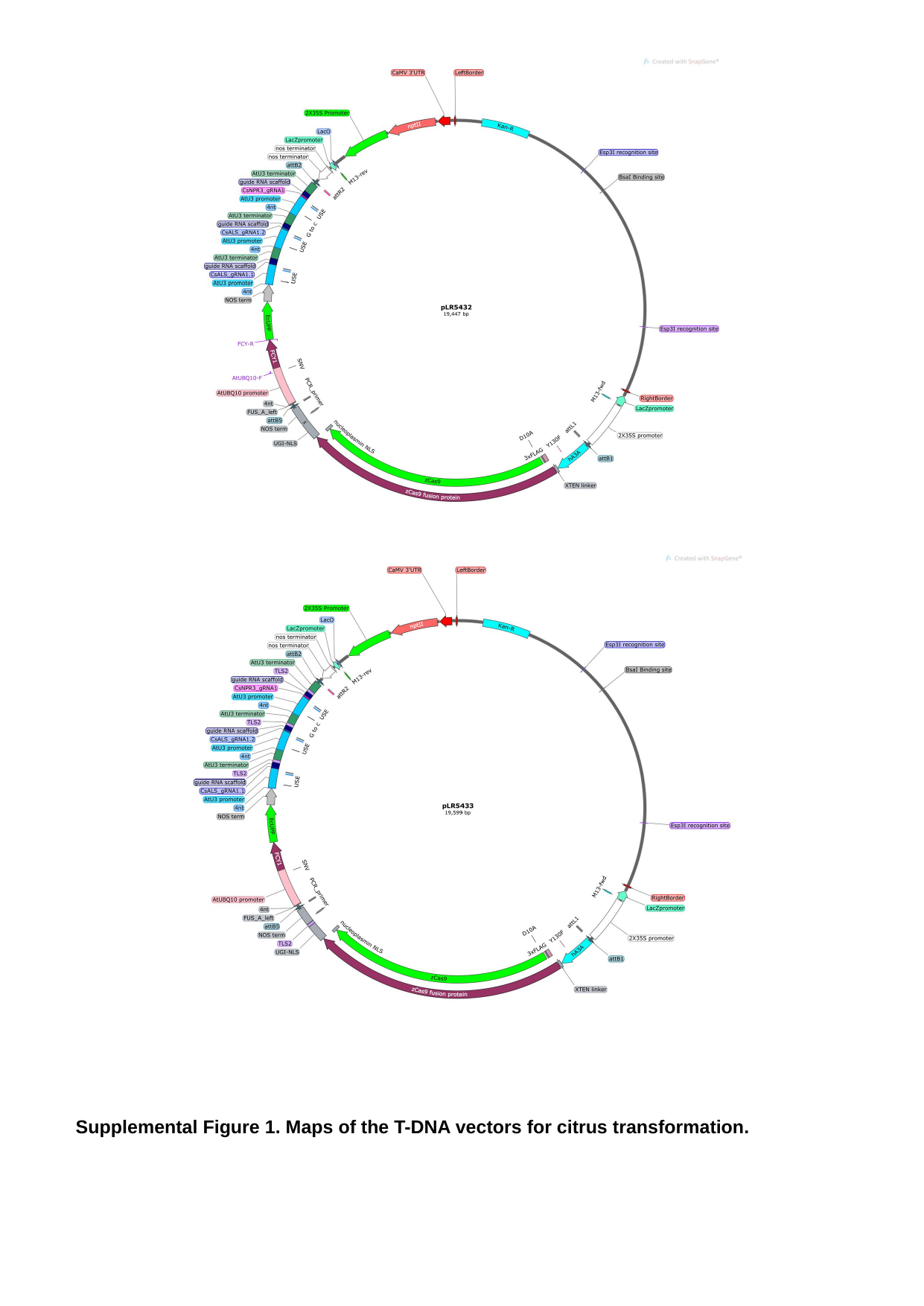

Supplemental Figure 1. Maps of the T-DNA vectors for citrus transformation.

## Slide 2
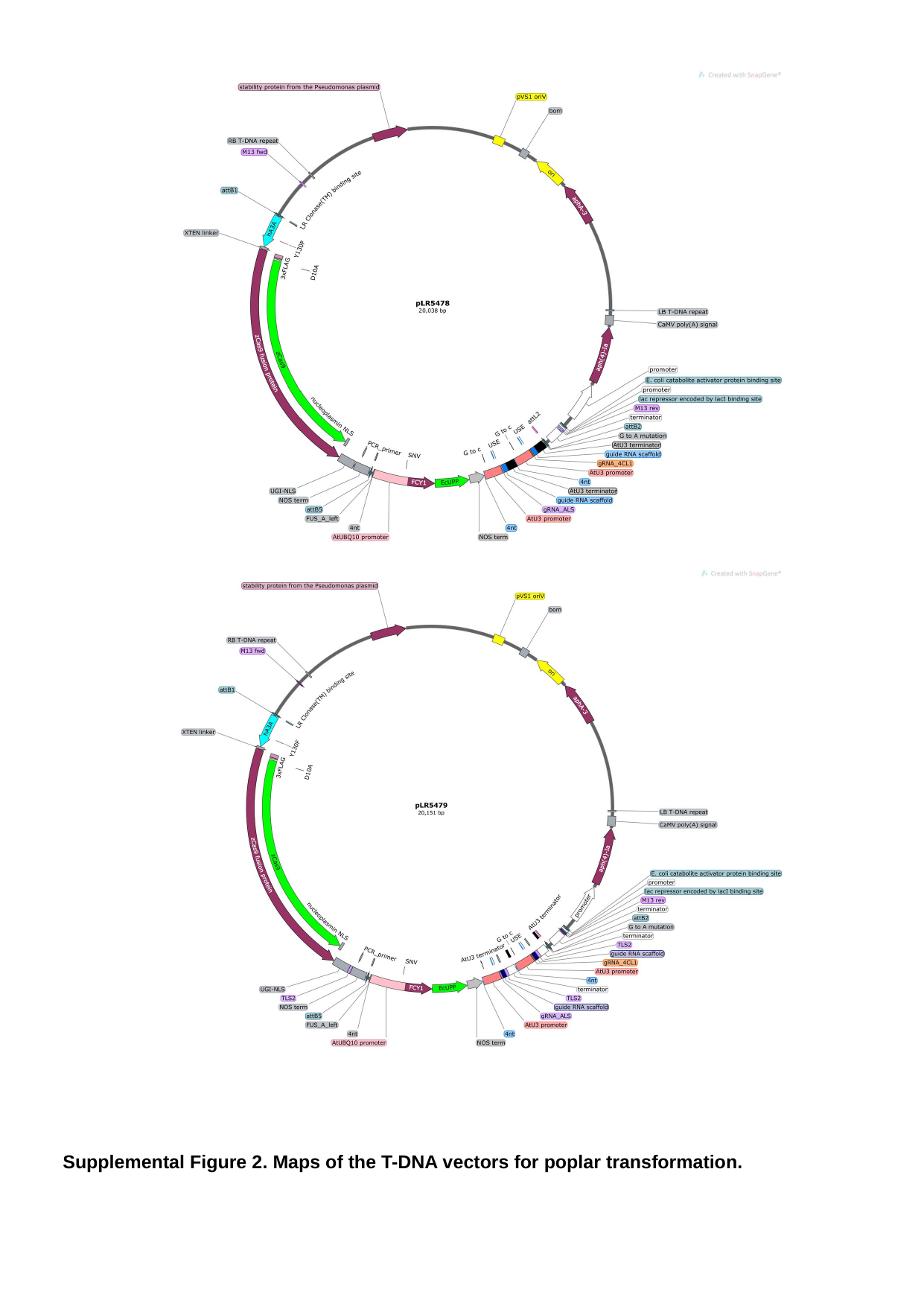

Supplemental Figure 2. Maps of the T-DNA vectors for poplar transformation.

## Slide 3
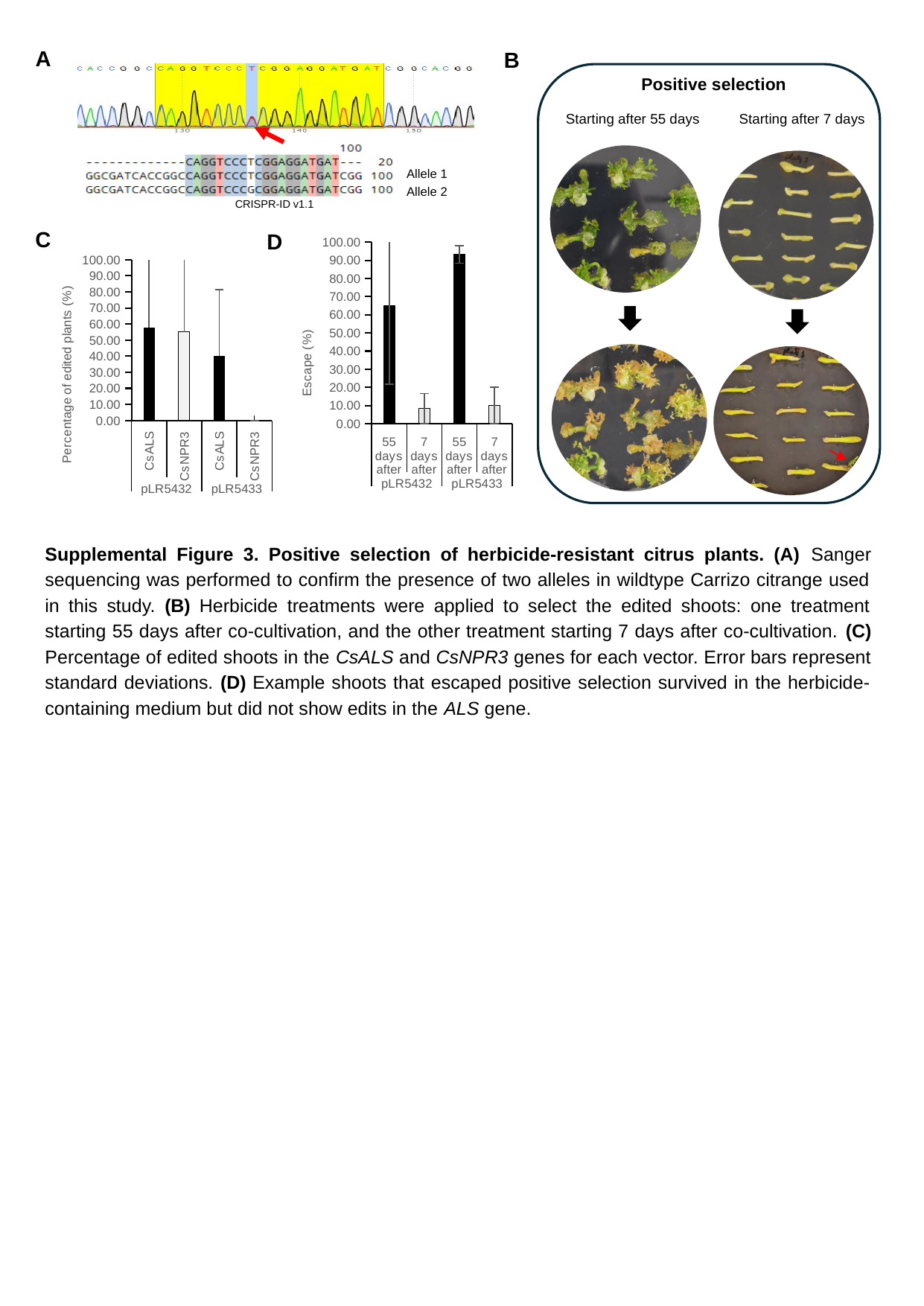

A
B
Positive selection
Starting after 7 days
Starting after 55 days
Allele 1
Allele 2
CRISPR-ID v1.1
C
D
### Chart
| Category | Escape (%) |
|---|---|
| 55 days after | 64.96969696969697 |
| 7 days after | 8.333333333333334 |
| 55 days after | 93.26599326599326 |
| 7 days after | 10.0 |
### Chart
| Category | Editing efficiency (%) |
|---|---|
| CsALS | 57.68484848484849 |
| CsNPR3 | 55.06666666666666 |
| CsALS | 40.04040404040404 |
| CsNPR3 | 0.0 |
Supplemental Figure 3. Positive selection of herbicide-resistant citrus plants. (A) Sanger sequencing was performed to confirm the presence of two alleles in wildtype Carrizo citrange used in this study. (B) Herbicide treatments were applied to select the edited shoots: one treatment starting 55 days after co-cultivation, and the other treatment starting 7 days after co-cultivation. (C) Percentage of edited shoots in the CsALS and CsNPR3 genes for each vector. Error bars represent standard deviations. (D) Example shoots that escaped positive selection survived in the herbicide-containing medium but did not show edits in the ALS gene.

## Slide 4
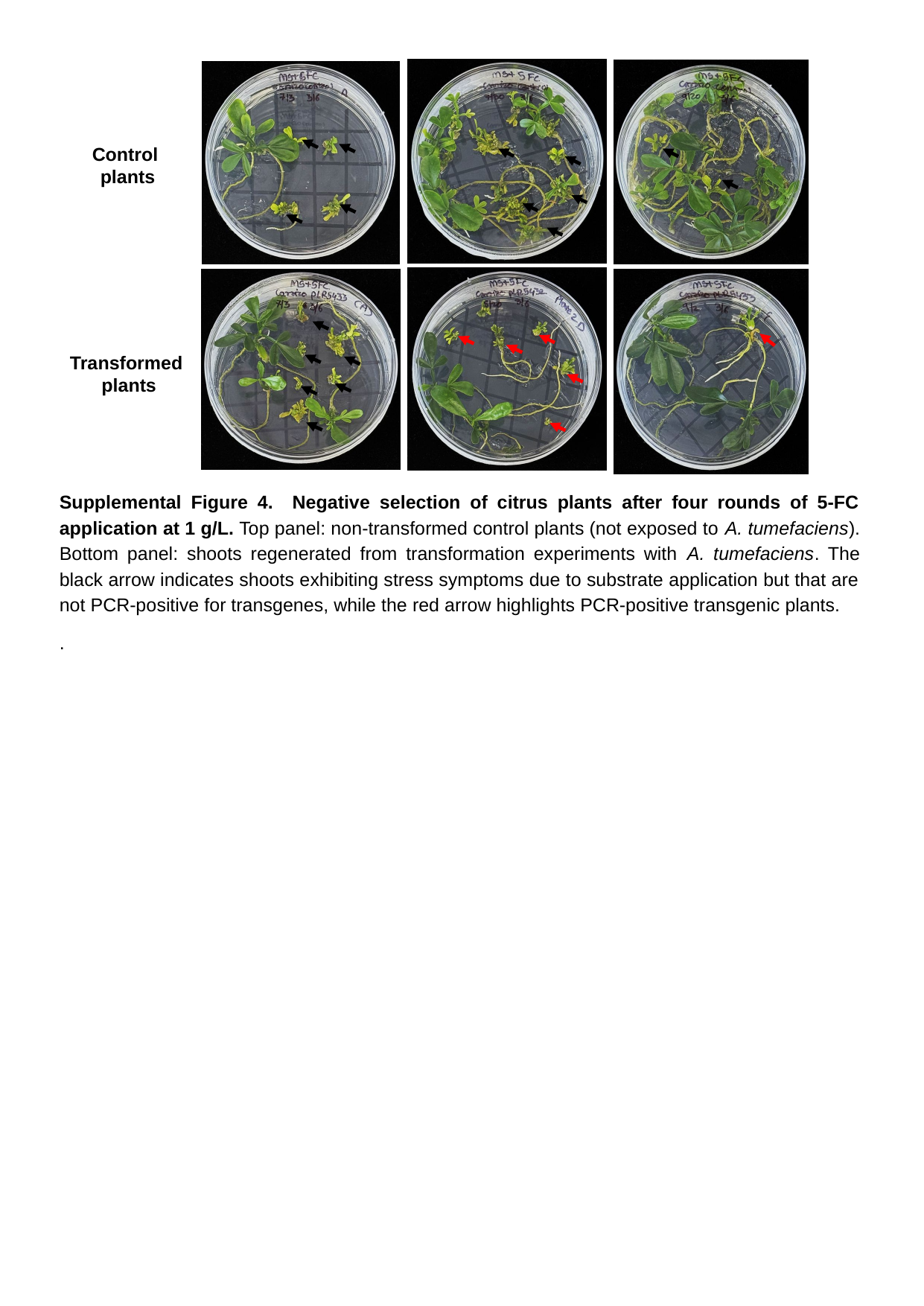

Control
plants
Transformed
 plants
Supplemental Figure 4. Negative selection of citrus plants after four rounds of 5-FC application at 1 g/L. Top panel: non-transformed control plants (not exposed to A. tumefaciens). Bottom panel: shoots regenerated from transformation experiments with A. tumefaciens. The black arrow indicates shoots exhibiting stress symptoms due to substrate application but that are not PCR-positive for transgenes, while the red arrow highlights PCR-positive transgenic plants.
.
