# Supplementary material for: Transgene-free genome editing in citrus and poplar trees using positive and negative selection markers
Source: Plant Cell Rep. 2025 Oct 22;44(11):244. doi: 10.1007/s00299-025-03627-2 (PMC12546424; doi:10.1007/s00299-025-03627-2)
Supplement: Supplementary file 2 — Supplementary file2 (DOCX 60 kb) [file 299_2025_3627_MOESM2_ESM.docx]

**Supplemental Table 1. Primers used for citrus.**

| **Function** | **Primers** | **Sequence 5'→3'** | **Amplicon (bp)** |
| --- | --- | --- | --- |
| **sgRNA assembly** | CsALS_gRNA1-1-F | GGTCACAGGTCCCTCGGAGGATGAT | **-** |
|  | CsALS_gRNA1-1-R | AAACATCATCCTCCGAGGGACCTGT | **-** |
|  | CsALS_gRNA1-2-F | GGTCACAGGTCCCGCGGAGGATGAT | **-** |
|  | CsALS_gRNA1-2-R | AAACATCATCCTCCGCGGGACCTGT | **-** |
|  | CsNPR3_gRNA1-F | GGTCATCATCGATGTATTTTGGCTGCA | **-** |
|  | CsNPR3_gRNA1-R | AAACTGCAGCCAAAATACATCGATGAT | **-** |
| **Vector assembly** | Linear_pYPQ265E2-F1 | GACGTCCGATCGTTCAAA | 8356 |
|  | Linear_pYPQ265E2-R1 | CTAAACCTTGCGTTTCTTC |  |
|  | TLS2-265E2-F1 | AGAAGAAACGCAAGGTTTAGATTATCAGAGTGGTGGTGGG | 86 |
|  | TLS2-265E2-R1 | TGTTTGAACGATCGGACGTCATCAGAGCGGACCTGTG |  |
|  | Linear_130-F3 | TGGAACTAGAATCTATAATGAGCCT | 3487 |
|  | Linear_130-R3 | ACTCGGTGCCACTTTTTC |  |
|  | TLS2-130-F2 | GAAAAAGTGGCACCGAGTCGGTGCATTATCAGAGTGGTGGTGGG | 306 |
|  | TLS2-130-R2 | AGGCTCATTATAGATTCTAGTTCCA |  |
| **Transgene detection** | AtUBQ10-F | GTTTCTAGTTTGTGCGATCGAATTTG | 523 |
|  | FCY-R | TATCTTCAAACCAATCCTGAGGTCTT |  |
|  | AtU3_NPR3-F | GGAGCACCATTGGTCATCATCGATGT | 328 |
|  | AttB2-R | AAGCTGGGTCGAATTCGCCCTTACTA |  |
|  | Cas9-FCY-F1 | TGGATATTAACAGGCTCAGCGATTA | 577 |
|  | Cas9-FCY-R1 | TTCCTCACATCGTAAACCTTGTAGT |  |
|  | Cas9-FCY-F2 | CGTCCAAGTATGTGAACTTCCTCTA | 586 |
|  | Cas9-FCY-R2 | CCTATAACTTCCTCGACTTCCTCAG |  |
|  | Kan-FCY-F1 | CGAGAAAGTATCCATCATGGCTGAT | 560 |
|  | Kan-FCY-R1 | CCTTATCTGGGAACTACTCACACATT |  |
| **Hi-TOM primers for first-round PCR** | ALS-HITOM-F1 | GGAGTGAGTACGGTGTGCAACCCGGCATCTGTATCGCC | 213 |
|  | ALS-HITOM-F2 | GAGTTGGATGCTGGATGGTGTCGTCAACATCAAGTACAAGAT |  |
|  | HITOM-CsNPR3-F1 | GGAGTGAGTACGGTGTGCACTCCTGATTGATTCCACTTGTGA | 211 |
|  | HITOM-CsNPR3-F1 | GAGTTGGATGCTGGATGGAAAGGCTTCATATCCAACCTTGC |  |

**Supplemental Table 2.** **Primers used for poplar.**

| **Function** | **Primers** | **Sequence 5'→3'** | **Amplicon (bp)** |
| --- | --- | --- | --- |
| **Poplar sgRNA assembly** | gRNA1_PtALS-F | GGTCACAGGTTCCGCGGCGAATGAT | **-** |
|  | gRNA1_PtALS-R | AAACATCATTCGCCGCGGAACCTGT | **-** |
|  | gRNA1_Pt4CL1-F | GGTCAAGTGTGGCTCAACAGGTAGA | **-** |
|  | gRNA1_Pt4CL1-R | AAACTCTACCTGTTGAGCCACACTT | **-** |
| **Vector assembly** | Linear_pYPQ265E2-F1 | GACGTCCGATCGTTCAAA | 8356 |
|  | Linear_pYPQ265E2-R1 | CTAAACCTTGCGTTTCTTC |  |
|  | TLS2-265E2-F1 | AGAAGAAACGCAAGGTTTAGATTATCAGAGTGGTGGTGGG | 86 |
|  | TLS2-265E2-R1 | TGTTTGAACGATCGGACGTCATCAGAGCGGACCTGTG |  |
|  | Linear_130-F3 | TGGAACTAGAATCTATAATGAGCCT | 3487 |
|  | Linear_130-R3 | ACTCGGTGCCACTTTTTC |  |
|  | TLS2-130-F2 | GAAAAAGTGGCACCGAGTCGGTGCATTATCAGAGTGGTGGTGGG | 306 |
|  | TLS2-130-R2 | AGGCTCATTATAGATTCTAGTTCCA |  |
| **Transgene detection** | TFD-Pt5478zCas9-F1 | GAAATGGCCCCTGAACTTAATCATGTG | 600 |
|  | TFD-Pt5478zCas9-R1 | GGGGATTACAAGGACCACGACATT |  |
|  | TFD-Pt5478EUPP-F1 | TTTCGTACCAAAGATTTTGTCACCG | 594 |
|  | TFD-Pt5478EUPP-R1 | GTCAAACACAAGCTGGGACTGATG |  |
| **Hi-TOM primers for poplar genes** | HiTOM-Pt4Cl1-F1 | GGAGTGAGTACGGTGTGCGATGTCGTAGCATTGCCTTATTCATC | 204 |
|  | HiTOM-Pt4Cl1-R1 | GAGTTGGATGCTGGATGGCAGACCACAGAGCATTATTGAATTCA |  |
|  | HiTOM-PtALS-F2 | GGAGTGAGTACGGTGTGCGTTTGTATTGCAACGTCAGGTCCTG | 212 |
|  | HiTOM-PtALS-R2 | GAGTTGGATGCTGGATGGAGGAATATCATCAACATCGAGAACCA |  |

**Supplemental Table 3. sgRNAs used in this study.**

| **Species** | **sgRNA name** | **Sequence 5'→3'** | **Insertion into the vectors** |
| --- | --- | --- | --- |
| Citrus | CsALS_gRNA1.1 | CAGGTCCCTCGGAGGATGAT | pYPQ132 and pYPQ132-TLS |
|  | CsALS_gRNA1.2 | CAGGTCCCGCGGAGGATGAT | pYPQ133 and pYPQ133-TLS |
|  | CsNPR3_sgRNA1 | TCATCGATGTATTTTGGCTGCA | pYPQ134 and pYPQ134-TLS |
| Poplar | PtALS_gRNA1 | CAGGTTCCGCGGCGAATGAT | pYPQ132 and pYPQ132-TLS |
|  | Pt4CL1_gRNA1 | AGTGTGGCTCAACAGGTAGA | pYPQ133 and pYPQ133-TLS |

**Supplemental Table 4. Comparison of co-editing strategies in citrus for generating edited and transgene-free plants.**

| **References** | **Citrus species** | **Base editing system** | **sgRNA expression** | **Genes** | **Herbicide resistance** | **Transgene detection method** | **Plants regenerated** | **Transgenic-free plants** | ***CsALS* edited plants** | **Escape (%)** | **Edited transgene-free plants (for both genes)** |
| --- | --- | --- | --- | --- | --- | --- | --- | --- | --- | --- | --- |
| Alquezar et al., 2022, Front Plant Sci | Carrizo | APOBEC1-nCas9-UGI | AtU6 | ALS | IMZ | PCR | 288 | 61 | 31 | 50.81 | - |
| Huang et al., 2022, Front Genome Ed | Carrizo | A3A-RAD51-DBD-nCas9-UGI | CsU6-tRNA | ALS | Chl | PCR and GFP | 3 | 2 | 2 | 66.66 | - |
| Huang et al., 2023, Nat Plants | Pummelo | Cas9-mPB and Cas12a | AtU6 | ALS and LOB1 | Chl | PCR and GFP | 107 | 103 | 8 | 92,52 | 1.9% (4) |
| Jia et al, 2024, Front Plant Sci | *C. sinensis* (Hamlin) | Cas9-mPB and Cas12a | AtU6 | ALS and LOB1 | Chl | PCR and GFP | 77 | 69 | 4 | 94,80 | 5.2% (4) |
| This study | Carrizo | hA3A-Y130F-znCas9-UGI | AtU3 | ALS and NPR3 | Chl | PCR and FCY-UPP | 97 | 72 | 51 | 42.32 | 23.75% (24) |
| This study | Carrizo | hA3A-Y130F-znCas9-UGI | AtU3; Mobile RNA tagged | ALS and NPR3 | Chl | PCR and FCY-UPP | 70 | 68 | 22 | 59.96 | 0% |

**Supplemental Table 5. Comparative analysis of co-editing strategies in Poplar for generating edited and transgene-free plants**

| **References** | **Poplar species** | **Base editing system** | **sgRNA cassette expression** | **Genes** | **Herbicide resistance** | **Transgene detection method** | **Plants regenerated** | **Transgenic-free plants** | ***PtALS* edited plants** | **Edited transgene-free plants (for both genes)** |
| --- | --- | --- | --- | --- | --- | --- | --- | --- | --- | --- |
| Hoengenaert et al., 2025, New Phytologist | 717 1B4 | A3A- nCas9-UGI | AtU6 | ALS and CCoAOMT1 | Chl | PCR and CodA | 37 | 18 | 37 | 7% |
| Wu et al., 2025, Plant Physiology | Shanxin | APOBEC3A-Y130F-nCas9-UGI | AtU3 | ALS and CEN | Chl | PCR and WGS | 45 | 4 | 45 | 26.7% |
| This study | 717 1B4 | hA3A-Y130F-znCas9-UGI | AtU3 | ALS and 4CL1 | Chl | PCR and FCY-UPP | 63 | 15 | 63 | 32.45% |
| This study | 7171B4 | hA3A-Y130F-znCas9-UGI | AtU3; Mobile RNA tagged | ALS and 4CL1 | Chl | PCR and FCY-UPP | 90 | 4 | 90 | 4.86% |

**Supplemental data 1.** Complete sequence of the citrus construct without TLS.

**Vector components legend:**

- Base editing system:2x35S promoter, ha3A, Cas9, UGI, NOS terminator.
- Toxin genes: AtUBQ10 promoter, ScFCY, UPP, NOS terminator.
- sgRNA: AtU3 promoter, CsALS_gRNA1.1,sgRNA scaffold, AtU3 terminator, AtU3 promoter, CsALS_gRNA1.2, sgRNA scaffold, AtU3 terminator, AtU3 promoter CsNPR3_sgRNA1, sgRNA scaffold, AtU3 terminator.
- The right and Left T-DNA borders are underlined.

>pLR5432 (19,447 bp) – (13,214 bp from T-DNA RB to LB)

5’-ccagccagccaacagctccccgaccggcagctcggcacaaaatcaccactcgatacaggcagcccatcagtccgggacggcgtcagcgggagagccgttgtaaggcggcagactttgctcatgttaccgatgctattcggaagaacggcaactaagctgccgggtttgaaacacggatgatctcgcggagggtagcatgttgattgtaacgatgacagagcgttgctgcctgtgatcaccgcggtttcaaaatcggctccgtcgatactatgttatacgccaactttgaaaacaactttgaaaaagctgttttctggtatttaaggttttagaatgcaaggaacagtgaattggagttcgtcttgttataattagcttcttggggtatctttaaatactgtagaaaagaggaaggaaataataaatggctaaaatgagaatatcaccggaattgaaaaaactgatcgaaaaataccgctgcgtaaaagatacggaaggaatgtctcctgctaaggtatataagctggtgggagaaaatgaaaacctatatttaaaaatgacggacagccggtataaagggaccacctatgatgtggaacgggaaaaggacatgatgctatggctggaaggaaagctgcctgttccaaaggtcctgcactttgaacggcatgatggctggagcaatctgctcatgagtgaggccgatggcgtcctttgctcggaagagtatgaagatgaacaaagccctgaaaagattatcgagctgtatgcggagtgcatcaggctctttcactccatcgacatatcggattgtccctatacgaatagcttagacagccgcttagccgaattggattacttactgaataacgatctggccgatgtggattgcgaaaactgggaagaagacactccatttaaagatccgcgcgagctgtatgattttttaaagacggaaaagcccgaagaggaacttgtcttttcccacggcgacctgggagacagcaacatctttgtgaaagatggcaaagtaagtggctttattgatcttgggagaagcggcagggcggacaagtggtatgacattgccttctgcgtccggtcgatcagggaggatatcggggaagaacagtatgtcgagctattttttgacttactggggatcaagcctgattgggagaaaataaaatattatattttactggatgaattgttttagtacctagaatgcatgaccaaaatcccttaacgtgagttttcgttccactgagcgtcagaccccgtagaaaagatcaaaggatcttcttgagatcctttttttctgcgcgtaatctgctgcttgcaaacaaaaaaaccaccgctaccagcggtggtttgtttgccggatcaagagctaccaactctttttccgaaggtaactggcttcagcagagcgcagataccaaatactgtccttctagtgtagccgtagttaggccaccacttcaagaactctgtagcaccgcctacatacctcgctctgctaatcctgttaccagtggctgctgccagtggcgataagtcgtgtcttaccgggttggactcaagacgatagttaccggataaggcgcagcggtcgggctgaacggggggttcgtgcacacagcccagcttggagcgaacgacctacaccgaactgagatacctacagcgtgagctatgagaaagcgccacgcttcccgaagggagaaaggcggacaggtatccggtaagcggcagggtcggaacaggagagcgcacgagggagcttccagggggaaacgcctggtatctttatagtcctgtcgggtttcgccacctctgacttgagcgtcgatttttgtgatgctcgtcaggggggcggagcctatggaaaaacgccagcaacgcggcctttttacggttcctggccttttgctggccttttgctcacatgttctttcctgcgttatcccctgattctgtggataaccgtattaccgcctttgagtgagctgataccgctcgccgcagccgaacgaccgagcgcagcgagtcagtgagcgaggaagcggaagagcgcctgatgcggtattttctccttacgcatctgtgcggtatttcacaccgcatatggtgcactctcagtacaatctgctctgatgccgcatagttaagccagtatacactccgctatcgctacgtgactgggtcatggctgcgccccgacacccgccaacacccgctgacgcgccctgacgggcttgtctgctcccggcatccgcttacagacaagctgtgaccgtctccgggagctgcatgtgtcagaggttttcaccgtcatcaccgaaacgcgcgaggcagggtgccttgatgtgggcgccggcggtcgagtggcgacggcgcggcttgtccgcgccctggtagattgcctggccgtaggccagccatttttgagcggccagcggccgcgataggccgacgcgaagcggcggggcgtagggagcgcagcgaccgaagggtaggcgctttttgcagctcttcggctgtgcgctggccagacagttatgcacaggccaggcgggttttaagagttttaataagttttaaagagttttaggcggaaaaatcgccttttttctcttttatatcagtcacttacatgtgtgaccggttcccaatgtacggctttgggttcccaatgtacgggttccggttcccaatgtacggctttgggttcccaatgtacgtgctatccacaggaaagagaccttttcgacctttttcccctgctagggcaatttgccctagcatctgctccgtacattaggaaccggcggatgcttcgccctcgatcaggttgcggtagcgcatgactaggatcgggccagcctgccccgcctcctccttcaaatcgtactccggcaggtcatttgacccgatcagcttgcgcacggtgaaacagaacttcttgaactctccggcgctgccactgcgttcgtagatcgtcttgaacaaccatctggcttctgccttgcctgcggcgcggcgtgccaggcggtagagaaaacggccgatgccgggatcgatcaaaaagtaatcggggtgaaccgtcagcacgtccgggttcttgccttctgtgatctcgcggtacatccaatcagctagctcgatctcgatgtactccggccgcccggtttcgctctttacgatcttgtagcggctaatcaaggcttcaccctcggataccgtcaccaggcggccgttcttggccttcttcgtacgctgcatggcaacgtgcgtggtgtttaaccgaatgcaggtttctaccaggtcgtctttctgctttccgccatcggctcgccggcagaacttgagtacgtccgcaacgtgtggacggaacacgcggccgggcttgtctcccttcccttcccggtatcggttcatggattcggttagatgggaaaccgccatcagtaccaggtcgtaatcccacacactggccatgccggccggccctgcggaaacctctacgtgcccgtctggaagctcgtagcggatcacctcgccagctcgtcggtcacgcttcgacagacggaaaacggccacgtccatgatgctgcgactatcgcgggtgcccacgtcatagagcatcggaacgaaaaaatctggttgctcgtcgcccttgggcggcttcctaatcgacggcgcaccggctgccggcggttgccgggattctttgcggattcgatcagcggccgcttgccacgattcaccggggcgtgcttctgcctcgatgcgttgccgctgggcggcctgcgcggccttcaacttctccaccaggtcatcacccagcgccgcgccgatttgtaccgggccggatggtttgcgaccgtcacgccgattcctcgggcttgggggttccagtgccattgcagggccggcagacaacccagccgcttacgcctggccaaccgcccgttcctccacacatggggcattccacggcgtcggtgcctggttgttcttgattttccatgccgcctcctttagccgctaaaattcatctactcatttattcatttgctcatttactctggtagctgcgcgatgtattcagatagcagctcggtaatggtcttgccttggcgtaccgcgtacatcttcagcttggtgtgatcctccgccggcaactgaaagttgacccgcttcatggctggcgtgtctgccaggctggccaacgttgcagccttgctgctgcgtgcgctcggacggccggcacttagcgtgtttgtgcttttgctcattttctctttacctcattaactcaaatgagttttgatttaatttcagcggccagcgcctggacctcgcgggcagcgtcgccctcgggttctgattcaagaacggttgtgccggcggcggcagtgcctgggtagctcacgcgctgcgtgatacgggactcaagaatgggcagctcgtacccggccagcgcctcggcaacctcaccgccgatgcgcgtgcctttgatcgcccgcgacacgacaaaggccgcttgtagccttccatccgtgacctcaatgcgctgcttaaccagctccaccaggtcggcggtggcccatatgtcgtaagggcttggctgcaccggaatcagcacgaagtcggctgccttgatcgcggacacagccaagtccgccgcctggggcgctccgtcgatcactacgaagtcgcgccggccgatggccttcacgtcgcggtcaatcgtcgggcggtcgatgccgacaacggttagcggttgatcttcccgcacggccgcccaatcgcgggcactgccctggggatcggaatcgactaacagaacatcggccccggcgagttgcagggcgcgggctagatgggttgcgatggtcgtcttgcctgacccgcctttctggttaagtacagcgataaccttcatgcgttccccttgcgtatttgtttatttactcatcgcatcatatacgcagcgaccgcatgacgcaagctgttttactcaaatacacatcacctttttagacggcggcgctcggtttcttcagcggccaagctggccggccaggccgccagcttggcatcagacaaaccggccaggatttcatgcagccgcacggttgagacgtgcgcgggcggctcgaacacgtacccggccgcgatcatctccgcctcgatctcttcggtaatgaaaaacggttcgtcctggccgtcctggtgcggtttcatgcttgttcctcttggcgttcattctcggcggccgccagggcgtcggcctcggtcaatgcgtcctcacggaaggcaccgcgccgcctggcctcggtgggcgtcacttcctcgctgcgctcaagtgcgcggtacagggtcgagcgatgcacgccaagcagtgcagccgcctctttcacggtgcggccttcctggtcgatcagctcgcgggcgtgcgcgatctgtgccggggtgagggtagggcgggggccaaacttcacgcctcgggccttggcggcctcgcgcccgctccgggtgcggtcgatgattagggaacgctcgaactcggcaatgccggcgaacacggtcaacaccatgcggccggccggcgtggtggtgtcggcccacggctctgccaggctacgcaggcccgcgccggcctcctggatgcgctcggcaatgtccagtaggtcgcgggtgctgcgggccaggcggtctagcctggtcactgtcacaacgtcgccagggcgtaggtggtcaagcatcctggccagctccgggcggtcgcgcctggtgccggtgatcttctcggaaaacagcttggtgcagccggccgcgtgcagttcggcccgttggttggtcaagtcctggtcgtcggtgctgacgcgggcatagcccagcaggccagcggcggcgctcttgttcatggcgtaatgtctccggttctagtcgcaagtattctactttatgcgactaaaacacgcgacaagaaaacgccaggaaaagggcagggcggcagcctgtcgcgtaacttaggacttgtgcgacatgtcgttttcagaagacggctgcactgaacgtcagaagccgactgcactatagcagcggaggggttggatcaaagtactttgatcccgaggggaaccctgtggttggcatgcacatacaaatggacgaacggataaaccttttcacgcccttttaaatatccgttattctaataaacgctcttttctcttaggtttacccgccaatatatcctgtcaaacactgatagtttaaactgaaggcgggaaacgacaatctgatccaagctcaagctgctctagcattcgccattcaggctgcgcaactgttgggaagggcgatcggtgcgggcctcttcgctattacgccagctggcgaaagggggatgtgctgcaaggcgattaagttgggtaacgccagggttttcccagtcacgacgttgtaaaacgacggccagtgccAAGCTTggcgtgcctgcaggtcaacatggtggagcacgacacacttgtctactccaaaaatatcaaagatacagtctcagaagaccaaagggcaattgagacttttcaacaaagggtaatatccggaaacctcctcggattccattgcccagctatctgtcactttattgtgaagatagtggaaaaggaaggtggctcctacaaatgccatcattgcgataaaggaaaggccatcgttgaagatgcctctgccgacagtggtcccaaagatggacccccacccacgaggagcatcgtggaaaaagaagacgttccaaccacgtcttcaaagcaagtggattgatgtgataacatggtggagcacgacacacttgtctactccaaaaatatcaaagatacagtctcagaagaccaaagggcaattgagacttttcaacaaagggtaatatccggaaacctcctcggattccattgcccagctatctgtcactttattgtgaagatagtggaaaaggaaggtggctcctacaaatgccatcattgcgataaaggaaaggccatcgttgaagatgcctctgccgacagtggtcccaaagatggacccccacccacgaggagcatcgtggaaaaagaagacgttccaaccacgtcttcaaagcaagtggattgatgtgatatctccactgacgtaagggatgacgcacaatcccactatccttcgcaagacccttcctctatataaggaagttcatttcatttggagaggacctcgactctagaggatccccgggtaccgggccccccctcgaggcgcgccaagctatcaaacaagtttgtacaaaaaagcaggctccgaattcgcccttcATGGAGGCCTCTCCGGCTTCAGGTCCTCGCCATCTCATGGACCCCCATATCTTTACCTCCAATTTCAATAACGGCATTGGCAGGCACAAGACATACCTCTGTTATGAGGTCGAGCGGCTCGATAACGGAACCTCAGTGAAGATGGACCAACACAGGGGTTTCCTTCATAACCAAGCGAAAAATCTCCTTTGCGGTTTCTATGGGCGGCACGCGGAGCTGAGATTCTTGGATTTGGTCCCGAGCCTCCAGCTGGATCCCGCGCAAATATACCGCGTCACTTGGTTCATATCATGGAGCCCGTGCTTTAGCTGGGGGTGTGCCGGTGAGGTTCGCGCGTTCCTCCAGGAAAACACCCATGTTCGCCTCCGGATTTTCGCAGCGAGGATTTtCGACTATGATCCACTTTACAAAGAGGCCCTCCAAATGCTTAGAGATGCTGGGGCTCAAGTTTCAATTATGACATACGACGAGTTTAAGCATTGTTGGGACACTTTTGTCGATCATCAGGGTTGTCCCTTCCAGCCGTGGGATGGTCTTGATGAGCATTCGCAAGCTTTGAGTGGCAGGCTTCGCGCGATACTTCAGAACCAGGGTAACtcaggttctgagacccctggcacaagtgagtcagcaacacccgagtccaccatggattacaaggaccacgacggggattacaaggaccacgacattgattacaaggatgatgatgacaagatggctccgaagaagaagaggaaggttggcatccacggggtgccagctgctgacaagaagtactcgatcggcctcgCtattgggactaactctgttggctgggccgtgatcaccgacgagtacaaggtgccctcaaagaagttcaaggtcctgggcaacaccgatcggcattccatcaagaagaatctcattggcgctctcctgttcgacagcggcgagacggctgaggctacgcggctcaagcgcaccgcccgcaggcggtacacgcgcaggaagaatcgcatctgctacctgcaggagattttctccaacgagatggcgaaggttgacgattctttcttccacaggctggaggagtcattcctcgtggaggaggataagaagcacgagcggcatccaatcttcggcaacattgtcgacgaggttgcctaccacgagaagtaccctacgatctaccatctgcggaagaagctcgtggactccacagataaggcggacctccgcctgatctacctcgctctggcccacatgattaagttcaggggccatttcctgatcgagggggatctcaacccggacaatagcgatgttgacaagctgttcatccagctcgtgcagacgtacaaccagctcttcgaggagaaccccattaatgcgtcaggcgtcgacgcgaaggctatcctgtccgctaggctctcgaagtctcggcgcctcgagaacctgatcgcccagctgccgggcgagaagaagaacggcctgttcgggaatctcattgcgctcagcctggggctcacgcccaacttcaagtcgaatttcgatctcgctgaggacgccaagctgcagctctccaaggacacatacgacgatgacctggataacctcctggcccagatcggcgatcagtacgcggacctgttcctcgctgccaagaatctgtcggacgccatcctcctgtctgatattctcagggtgaacaccgagattacgaaggctccgctctcagcctccatgatcaagcgctacgacgagcaccatcaggatctgaccctcctgaaggcgctggtcaggcagcagctccccgagaagtacaaggagatcttcttcgatcagtcgaagaacggctacgctgggtacattgacggcggggcctctcaggaggagttctacaagttcatcaagccgattctggagaagatggacggcacggaggagctgctggtgaagctcaatcgcgaggacctcctgaggaagcagcggacattcgataacggcagcatcccacaccagattcatctcggggagctgcacgctatcctgaggaggcaggaggacttctaccctttcctcaaggataaccgcgagaagatcgagaagattctgactttcaggatcccgtactacgtcggcccactcgctaggggcaactcccgcttcgcttggatgacccgcaagtcagaggagacgatcacgccgtggaacttcgaggaggtggtcgacaagggcgctagcgctcagtcgttcatcgagaggatgacgaatttcgacaagaacctgccaaatgagaaggtgctccctaagcactcgctcctgtacgagtacttcacagtctacaacgagctgactaaggtgaagtatgtgaccgagggcatgaggaagccggctttcctgtctggggagcagaagaaggccatcgtggacctcctgttcaagaccaaccggaaggtcacggttaagcagctcaaggaggactacttcaagaagattgagtgcttcgattcggtcgagatctctggcgttgaggaccgcttcaacgcctccctggggacctaccacgatctcctgaagatcattaaggataaggacttcctggacaacgaggagaatgaggatatcctcgaggacattgtgctgacactcactctgttcgaggaccgggagatgatcgaggagcgcctgaagacttacgcccatctcttcgatgacaaggtcatgaagcagctcaagaggaggaggtacaccggctgggggaggctgagcaggaagctcatcaacggcattcgggacaagcagtccgggaagacgatcctcgacttcctgaagagcgatggcttcgcgaaccgcaatttcatgcagctgattcacgatgacagcctcacattcaaggaggatatccagaaggctcaggtgagcggccagggggactcgctgcacgagcatatcgcgaacctcgctggctcgccagctatcaagaaggggattctgcagaccgtgaaggttgtggacgagctggtgaaggtcatgggcaggcacaagcctgagaacatcgtcattgagatggcccgggagaatcagaccacgcagaagggccagaagaactcacgcgagaggatgaagaggatcgaggagggcattaaggagctggggtcccagatcctcaaggagcacccggtggagaacacgcagctgcagaatgagaagctctacctgtactacctccagaatggccgcgatatgtatgtggaccaggagctggatattaacaggctcagcgattacgacgtcgatcatatcgttccacagtcattcctgaaggatgactccattgacaacaaggtcctcaccaggtcggacaagaaccggggcaagtctgataatgttccttcagaggaggtcgttaagaagatgaagaactactggcgccagctcctgaatgccaagctgatcacgcagcggaagttcgataacctcacaaaggctgagaggggcgggctctctgagctggacaaggcgggcttcatcaagaggcagctggtcgagacacggcagatcactaagcacgttgcgcagattctcgactcacggatgaacactaagtacgatgagaatgacaagctgatccgcgaggtgaaggtcatcaccctgaagtcaaagctcgtctccgacttcaggaaggatttccagttctacaaggttcgggagatcaacaattaccaccatgcccatgacgcgtacctgaacgcggtggtcggcacagctctgatcaagaagtacccaaagctcgagagcgagttcgtgtacggggactacaaggtttacgatgtgaggaagatgatcgccaagtcggagcaggagattggcaaggctaccgccaagtacttcttctactctaacattatgaatttcttcaagacagagatcactctggccaatggcgagatccggaagcgccccctcatcgagacgaacggcgagacgggggagatcgtgtgggacaagggcagggatttcgcgaccgtcaggaaggttctctccatgccacaagtgaatatcgtcaagaagacagaggtccagactggcgggttctctaaggagtcaattctgcctaagcggaacagcgacaagctcatcgcccgcaagaaggactgggatccgaagaagtacggcgggttcgacagccccactgtggcctactcggtcctggttgtggcgaaggttgagaagggcaagtccaagaagctcaagagcgtgaaggagctgctggggatcacgattatggagcgctccagcttcgagaagaacccgatcgatttcctggaggcgaagggctacaaggaggtgaagaaggacctgatcattaagctccccaagtactcactcttcgagctggagaacggcaggaagcggatgctggcttccgctggcgagctgcagaaggggaacgagctggctctgccgtccaagtatgtgaacttcctctacctggcctcccactacgagaagctcaagggcagccccgaggacaacgagcagaagcagctgttcgtcgagcagcacaagcattacctcgacgagatcattgagcagatttccgagttctccaagcgcgtgatcctggccgacgcgaatctggataaggtcctctccgcgtacaacaagcaccgcgacaagccaatcagggagcaggctgagaatatcattcatctcttcaccctgacgaacctcggcgcccctgctgctttcaagtacttcgacacaactatcgatcgcaagaggtacacaagcactaaggaggtcctggacgcgaccctcatccaccagtcgattaccggcctctacgagacgcgcatcgacctgtctcagctcgggggcgacaagcggccagcggcgacgaagaaggcggggcaggcgaagaagaagaagggagacggatcaggcgggtcaacaaacctcagtgacatcatagagaaggaaactggtaagcaactggttattcaagagagcattctgatgctccctgaggaagtcgaggaagttataggaaacaagcctgagagcgatatactcgtgcatacggcgtatgacgagagcacggatgaaaatgtgatgctcttgaccagcgatgcgccagaatataaaccctgggcactggttattcaggactctaacggggaaaataaaatcaagatgctctcaggtggctccccaaagaagaaacgcaaggtttagtggcggccgccgacgtccgatcgttcaaacatttggcaataaagtttcttaagattgaatcctgttgccggtcttgcgatgattatcatataatttctgttgaattacgttaagcatgtaataattaacatgtaatgcatgacgttatttatgagatgggtttttatgattagagtcccgcaattatacatttaatacgcgatagaaaacaaaatatagcgcgcaaactaggataaattatcgcgcgcggtgtcatctatgttactagatcgggaattgatcccccctcgacagcttccggaaagggcgaattcgcaactttgtatacaaaagttgccccatggcgttccctctagataacgcaggatccccaagtggtggctattcgacgagtcagtaataaacggcgtcaaagtggttgcagccggcacacacgagtcgtgtttatcaactcaaagcacaaatacttttcctcaacctaaaaataaggcaattagccaaaaacaactttgcgtgtaaacaacgctcaatacacgtgtcattttattattagctattgcttcaccgccttagctttctcgtgacctagtcgtcctcgtcttttcttcttcttcttctataaaacaatacccaaagagctcttcttcttcacaattcagatttcaatttctcaaaatcttaaaaactttctctcaattctctctaccgtgatcaaggtaaatttctgtgttccttattctctcaaaatcttcgattttgttttcgttcgatcccaatttcgtatatgttctttggtttagattctgttaatcttagatcgaagacgattttctgggtttgatcgttagatatcatcttaattctcgattagggtttcatagatatcatccgatttgttcaaataatttgagttttgtcgaataattactcttcgatttgtgatttctatGtagatctggtgttagtttctagtttgtgcgatcgaatttgtcgattaatctgagtttttctgattaacagAATGGTGACAGGGGGAATGGCAAGCAAGTGGGATCAGAAGGGTATGGACATTGCCTATGAGGAGGCGGCCTTAGGTTACAAAGAGGGTGGTGTTCCTATTGGCGGATGTCTTATCAATAACAAAGACGGAAGTGTTCTCGGTCGTGGTCACAACATGAGATTTCAAAAGGGATCCGCCACACTACATGGTGAGATCTCCACTTTGGAAAACTGTGGGAGATTAGAGGGCAAAGTGTACAAAGATACCACTTTGTATACGACGCTGTCTCCATGCGACATGTGTACAGGTGCCATCATCATGTATGGTATTCCACGCTGTGTTGTCGGTGAGAACGTTAATTTCAAAAGTAAGGGCGAGAAATATTTACAAACTAGAGGTCACGAGGTTGTTGTTGTTGACGATGAGAGGTGTAAAAAGATCATGAAACAATTTATCGATGAAAGACCTCAGGATTGGTTTGAAGATATTGGTGAGgctTCCGccAAGATCGTGGAAGTCAAACACCCACTCGTCAAACACAAGCTGGGACTGATGCGTGAGCAAGATATCAGCACCAAGCGCTTTCGCGAACTCGCTTCCGAAGTGGGTAGCCTGCTGACTTACGAAGCGACCGCCGACCTCGAAACGGAAAAAGTAACTATCGAAGGCTGGAACGGCCCGGTAGAAATCGACCAGATCAAAGGTAAGAAAATTACCGTTGTGCCAATTCTGCGTGCGGGTCTTGGTATGATGGACGGTGTGCTGGAAAACGTTCCGAGCGCGCGCATCAGCGTTGTCGGTATGTACCGTAATGAAGAAACGCTGGAGCCGGTACCGTACTTCCAGAAACTGGTTTCTAACATCGATGAGCGTATGGCGCTGATCGTTGACCCAATGCTGGCAACCGGTGGTTCCGTTATCGCGACCATCGACCTGCTGAAAAAAGCGGGCTGCAGCAGCATCAAAGTTCTGGTGCTGGTAGCTGCGCCAGAAGGTATCGCTGCGCTGGAAAAAGCGCACCCGGACGTCGAACTGTATACCGCATCGATTGATCAGGGACTGAACGAGCACGGATACATTATTCCGGGCCTCGGCGATGCCGGTGACAAAATCTTTGGTACGAAAtaGccgatcgttcaaacatttggcaataaagtttcttaagattgaatcctgttgccggtcttgcgatgattatcatataatttctgttgaattacgttaagcatgtaataattaacatgtaatgcatgacgttatttatgagatgggtttttatgattagagtcccgcaattatacatttaatacgcgatagaaaacaaaatatagcgcgcaaactaggataaattatcgcgcgcggtgtcatctatgttactagatcgggaattgatcccccctcgacagcatgTTTACTTTAAATTTTTCTTATGGCTCAGCCTGTGATGGATAACTGAATCAAACAAATGGCGTCTGGGTTTAAGAAcATCTGTTTTGGCTATGTTGGACGAAACAAGTGAACTTTTAGGATCAACTTCAGTTTATATACGGAGCTTATATCGAGCAATAAGATAAGTGGGCTTTTTATGTAATTTAATGGGCTATCGTCCATATATTCACTAATACCCATGCCCAGTACCCATGTATGCGTTTCATATAAGCTCCTAATTTCTCCCACATCGCTCAAATCTAAACAAATCTTGTTGTATATATAACACTGAGGGAGCACCATTGGTCACAGGTCCCTCGGAGGATGATGTTTTAGAGCTAGAAATAGCAAGTTAAAATAAGGCTAGTCCGTTATCAACTTGAAAAAGTGGCACCGAGTCGGTGCTTTTTTTTCCCTTTCCTTTTTTCTTTTTTTTGCCATAAACTTAAATTTGTATATCGATCATTGTAGATATTGAAAACCTAGAACAAACCAACATCCATGTGAATGTCTTTCATGACTGATTTAGAGATAATTCTTGAATTTTGGAACTAGAATCTATAATGAGCCTaaattaaaacattgtgggacTTTACTTTAAATTTTTCTTATGGCTCAGCCTGTGATGGATAACTGAATCAAACAAATGGCGTCTGGGTTTAAGAAcATCTGTTTTGGCTATGTTGGACGAAACAAGTGAACTTTTAGGATCAACTTCCGTTTATATACGGAGCTTATATCGAGCAATAAGATAAGTGGGCTTTTTATGTAATTTAATGGGCTATCGTCCATATATTCACTAATACCCATGCCCAGTACCCATGTATGCGTTTCATATAAGCTCCTAATTTCTCCCACATCGCTCAAATCTAAACAAATCTTGTTGTATATATAACACTGAGGGAGCACCATTGGTCACAGGTCCCGCGGAGGATGATGTTTTAGAGCTAGAAATAGCAAGTTAAAATAAGGCTAGTCCGTTATCAACTTGAAAAAGTGGCACCGAGTCGGTGCTTTTTTTTCCCTTTCCTTTTTTCTTTTTTTTGCCATAAACTTAAATTTGTATATCGATCATTGTAGATATTGAAAACCTAGAACAAACCAACATCCATGTGAATGTCTTTCATGACTGATTTAGAGATAATTCTTGAATTTTGGAACTAGAATCTATAATGAGCCTaaattaaaacattgtgccagTTTACTTTAAATTTTTCTTATGGCTCAGCCTGTGATGGATAACTGAATCAAACAAATGGCGTCTGGGTTTAAGAAcATCTGTTTTGGCTATGTTGGACGAAACAAGTGAACTTTTAGGATCAACTTCCGTTTATATACGGAGCTTATATCGAGCAATAAGATAAGTGGGCTTTTTATGTAATTTAATGGGCTATCGTCCATATATTCACTAATACCCATGCCCAGTACCCATGTATGCGTTTCATATAAGCTCCTAATTTCTCCCACATCGCTCAAATCTAAACAAATCTTGTTGTATATATAACACTGAGGGAGCACCATTGGTCATCATCGATGTATTTTGGCTGCAGTTTTAGAGCTAGAAATAGCAAGTTAAAATAAGGCTAGTCCGTTATCAACTTGAAAAAGTGGCACCGAGTCGGTGCTTTTTTTTCCCTTTCCTTTTTTCTTTTTTTTGCCATAAACTTAAATTTGTATATCGATCATTGTAGATATTGAAAACCTAGAACAAACCAACATCCATGTGAATGTCTTTCATGACTGATTTAGAGATAATTCTTGAATTTTGGAACTAGAATCTATAATGAGCCTaaattaaaacattgtgTGTTactagtaagggcgaattcgacccagctttcttgtacaaagtggttcgataattcttaattaactagttctagagcggccgccaccgcggtggagctcgaatttccccgatcgttcaaacatttggcaataaagtttcttaagattgaatcctgttgccggtcttgcgatgattatcatataatttctgttgaattacgttaagcatgtaataattaacatgtaatgcatgacgttatttatgagatgggtttttatgattagagtcccgcaattatacatttaatacgcgatagaaaacaaaatatagcgcgcaaactaggataaattatcgcgcgcggtgtcatctatgttactgaattcgtaatcatggtcatagAAGCTTgcatgcctgcaggtcgactctagaggatccccgggtaccgagctcgaattcgtaatcatgtcatagctgtttcctgtgtgaaattgttatccgctcacaattccacacaacatacgagccggaagcataaagtgtaaagcctggggtgcctaatgagtgagctaactcacattaattgcgttgcgctcactgcccgctttccagtcgggaaacctgtcgtgccagctgcattaatgaatcggccaacgcgcggggagaggcggtttgcgtattggctagagcagcttgccaacatggtggagcacgacactctcgtctactccaagaatatcaaagatacagtctcagaagaccaaagggctattgagacttttcaacaaagggtaatatcgggaaacctcctcggattccattgcccagctatctgtcacttcatcaaaaggacagtagaaaaggaaggtggcacctacaaatgccatcattgcgataaaggaaaggctatcgttcaagatgcctctgccgacagtggtcccaaagatggacccccacccacgaggagcatcgtggaaaaagaagacgttccaaccacgtcttcaaagcaagtggattgatgtgataacatggtggagcacgacactctcgtctactccaagaatatcaaagatacagtctcagaagaccaaagggctattgagacttttcaacaaagggtaatatcgggaaacctcctcggattccattgcccagctatctgtcacttcatcaaaaggacagtagaaaaggaaggtggcacctacaaatgccatcattgcgataaaggaaaggctatcgttcaagatgcctctgccgacagtggtcccaaagatggacccccacccacgaggagcatcgtggaaaaagaagacgttccaaccacgtcttcaaagcaagtggattgatgtgatatctccactgacgtaagggatgacgcacaatcccactatccttcgcaagaccttcctctatataaggaagttcatttcatttggagaggacacgctgaaatcaccagtctctctctacaaatctatctctctcgagctttcgcagatctgtcgatcgaccatggggattgaacaagatggattgcacgcaggttctccggccgcttgggtggagaggctattcggctatgactgggcacaacagacaatcggctgctctgatgccgccgtgttccggctgtcagcgcaggggcgcccggttctttttgtcaagaccgacctgtccggtgccctgaatgaactccaggacgaggcagcgcggctatcgtggctggccacgacgggcgttccttgcgcagctgtgctcgacgttgtcactgaagcgggaagggactggctgctattgggcgaagtgccggggcaggatctcctgtcatctcaccttgctcctgccgagaaagtatccatcatggctgatgcaatgcggcggctgcatacgcttgatccggctacctgcccattcgaccaccaagcgaaacatcgcatcgagcgagcacgtactcggatggaagccggtcttgtcgatcaggatgatctggacgaagagcatcaggggctcgcgccagccgaactgttcgccaggctcaaggcgcgcatgcccgacggcgaggatctcgtcgtgacacatggcgatgcctgcttgccgaatatcatggtggaaaatggccgcttttctggattcatcgactgtggccggctgggtgtggcggaccgctatcaggacatagcgttggctacccgtgatattgctgaagagcttggcggcgaatgggctgaccgcttccTcgtgctttacggtatcgccgctcccgattcgcagcgcatcgccttctatcgccttcttgacgagttcttctgagcgggactctggggttcggatcgatcctctagctagagtcgatcgacaagctcgagtttctccataataatgtgtgagtagttcccagataagggaattagggttcctatagggtttcgctcatgtgttgagcatataagaaacccttagtatgtatttgtatttgtaaaatacttctatcaataaaatttctaattcctaaaaccaaaatccagtactaaaatccagatcccccgaattaattcggcgttaattcagtacattaaaaacgtccgcaatgtgttattaagttgtctaagcgtcaatttgtttacaccacaatatatcctgcca-3’

**Supplemental data 2.** Complete sequence of the citrus construct with TLS.

**Vector components legend:**

- Base editing system:2x35S promoter, ha3A, Cas9, UGI, TLS sequence, NOS terminator.
- Toxin genes: AtUBQ10 promoter, ScFCY, UPP, NOS terminator.
- sgRNA: AtU3 promoter, CsALS_gRNA1.1, sgRNA scaffold, TLS sequence, AtU3 terminator, AtU3 promoter, CsALS_gRNA1.2, sgRNA scaffold, TLS sequence, AtU3 terminator, AtU3 promoter, CsNPR3_sgRNA1, sgRNA scaffold, TLS sequence, AtU3 terminator.
- The right and eft T-DNA borders are underlined.

>pLR5433 (19,599 bp) – (13,366 bp for T-DNA RB to LB)

ccagccagccaacagctccccgaccggcagctcggcacaaaatcaccactcgatacaggcagcccatcagtccgggacggcgtcagcgggagagccgttgtaaggcggcagactttgctcatgttaccgatgctattcggaagaacggcaactaagctgccgggtttgaaacacggatgatctcgcggagggtagcatgttgattgtaacgatgacagagcgttgctgcctgtgatcaccgcggtttcaaaatcggctccgtcgatactatgttatacgccaactttgaaaacaactttgaaaaagctgttttctggtatttaaggttttagaatgcaaggaacagtgaattggagttcgtcttgttataattagcttcttggggtatctttaaatactgtagaaaagaggaaggaaataataaatggctaaaatgagaatatcaccggaattgaaaaaactgatcgaaaaataccgctgcgtaaaagatacggaaggaatgtctcctgctaaggtatataagctggtgggagaaaatgaaaacctatatttaaaaatgacggacagccggtataaagggaccacctatgatgtggaacgggaaaaggacatgatgctatggctggaaggaaagctgcctgttccaaaggtcctgcactttgaacggcatgatggctggagcaatctgctcatgagtgaggccgatggcgtcctttgctcggaagagtatgaagatgaacaaagccctgaaaagattatcgagctgtatgcggagtgcatcaggctctttcactccatcgacatatcggattgtccctatacgaatagcttagacagccgcttagccgaattggattacttactgaataacgatctggccgatgtggattgcgaaaactgggaagaagacactccatttaaagatccgcgcgagctgtatgattttttaaagacggaaaagcccgaagaggaacttgtcttttcccacggcgacctgggagacagcaacatctttgtgaaagatggcaaagtaagtggctttattgatcttgggagaagcggcagggcggacaagtggtatgacattgccttctgcgtccggtcgatcagggaggatatcggggaagaacagtatgtcgagctattttttgacttactggggatcaagcctgattgggagaaaataaaatattatattttactggatgaattgttttagtacctagaatgcatgaccaaaatcccttaacgtgagttttcgttccactgagcgtcagaccccgtagaaaagatcaaaggatcttcttgagatcctttttttctgcgcgtaatctgctgcttgcaaacaaaaaaaccaccgctaccagcggtggtttgtttgccggatcaagagctaccaactctttttccgaaggtaactggcttcagcagagcgcagataccaaatactgtccttctagtgtagccgtagttaggccaccacttcaagaactctgtagcaccgcctacatacctcgctctgctaatcctgttaccagtggctgctgccagtggcgataagtcgtgtcttaccgggttggactcaagacgatagttaccggataaggcgcagcggtcgggctgaacggggggttcgtgcacacagcccagcttggagcgaacgacctacaccgaactgagatacctacagcgtgagctatgagaaagcgccacgcttcccgaagggagaaaggcggacaggtatccggtaagcggcagggtcggaacaggagagcgcacgagggagcttccagggggaaacgcctggtatctttatagtcctgtcgggtttcgccacctctgacttgagcgtcgatttttgtgatgctcgtcaggggggcggagcctatggaaaaacgccagcaacgcggcctttttacggttcctggccttttgctggccttttgctcacatgttctttcctgcgttatcccctgattctgtggataaccgtattaccgcctttgagtgagctgataccgctcgccgcagccgaacgaccgagcgcagcgagtcagtgagcgaggaagcggaagagcgcctgatgcggtattttctccttacgcatctgtgcggtatttcacaccgcatatggtgcactctcagtacaatctgctctgatgccgcatagttaagccagtatacactccgctatcgctacgtgactgggtcatggctgcgccccgacacccgccaacacccgctgacgcgccctgacgggcttgtctgctcccggcatccgcttacagacaagctgtgaccgtctccgggagctgcatgtgtcagaggttttcaccgtcatcaccgaaacgcgcgaggcagggtgccttgatgtgggcgccggcggtcgagtggcgacggcgcggcttgtccgcgccctggtagattgcctggccgtaggccagccatttttgagcggccagcggccgcgataggccgacgcgaagcggcggggcgtagggagcgcagcgaccgaagggtaggcgctttttgcagctcttcggctgtgcgctggccagacagttatgcacaggccaggcgggttttaagagttttaataagttttaaagagttttaggcggaaaaatcgccttttttctcttttatatcagtcacttacatgtgtgaccggttcccaatgtacggctttgggttcccaatgtacgggttccggttcccaatgtacggctttgggttcccaatgtacgtgctatccacaggaaagagaccttttcgacctttttcccctgctagggcaatttgccctagcatctgctccgtacattaggaaccggcggatgcttcgccctcgatcaggttgcggtagcgcatgactaggatcgggccagcctgccccgcctcctccttcaaatcgtactccggcaggtcatttgacccgatcagcttgcgcacggtgaaacagaacttcttgaactctccggcgctgccactgcgttcgtagatcgtcttgaacaaccatctggcttctgccttgcctgcggcgcggcgtgccaggcggtagagaaaacggccgatgccgggatcgatcaaaaagtaatcggggtgaaccgtcagcacgtccgggttcttgccttctgtgatctcgcggtacatccaatcagctagctcgatctcgatgtactccggccgcccggtttcgctctttacgatcttgtagcggctaatcaaggcttcaccctcggataccgtcaccaggcggccgttcttggccttcttcgtacgctgcatggcaacgtgcgtggtgtttaaccgaatgcaggtttctaccaggtcgtctttctgctttccgccatcggctcgccggcagaacttgagtacgtccgcaacgtgtggacggaacacgcggccgggcttgtctcccttcccttcccggtatcggttcatggattcggttagatgggaaaccgccatcagtaccaggtcgtaatcccacacactggccatgccggccggccctgcggaaacctctacgtgcccgtctggaagctcgtagcggatcacctcgccagctcgtcggtcacgcttcgacagacggaaaacggccacgtccatgatgctgcgactatcgcgggtgcccacgtcatagagcatcggaacgaaaaaatctggttgctcgtcgcccttgggcggcttcctaatcgacggcgcaccggctgccggcggttgccgggattctttgcggattcgatcagcggccgcttgccacgattcaccggggcgtgcttctgcctcgatgcgttgccgctgggcggcctgcgcggccttcaacttctccaccaggtcatcacccagcgccgcgccgatttgtaccgggccggatggtttgcgaccgtcacgccgattcctcgggcttgggggttccagtgccattgcagggccggcagacaacccagccgcttacgcctggccaaccgcccgttcctccacacatggggcattccacggcgtcggtgcctggttgttcttgattttccatgccgcctcctttagccgctaaaattcatctactcatttattcatttgctcatttactctggtagctgcgcgatgtattcagatagcagctcggtaatggtcttgccttggcgtaccgcgtacatcttcagcttggtgtgatcctccgccggcaactgaaagttgacccgcttcatggctggcgtgtctgccaggctggccaacgttgcagccttgctgctgcgtgcgctcggacggccggcacttagcgtgtttgtgcttttgctcattttctctttacctcattaactcaaatgagttttgatttaatttcagcggccagcgcctggacctcgcgggcagcgtcgccctcgggttctgattcaagaacggttgtgccggcggcggcagtgcctgggtagctcacgcgctgcgtgatacgggactcaagaatgggcagctcgtacccggccagcgcctcggcaacctcaccgccgatgcgcgtgcctttgatcgcccgcgacacgacaaaggccgcttgtagccttccatccgtgacctcaatgcgctgcttaaccagctccaccaggtcggcggtggcccatatgtcgtaagggcttggctgcaccggaatcagcacgaagtcggctgccttgatcgcggacacagccaagtccgccgcctggggcgctccgtcgatcactacgaagtcgcgccggccgatggccttcacgtcgcggtcaatcgtcgggcggtcgatgccgacaacggttagcggttgatcttcccgcacggccgcccaatcgcgggcactgccctggggatcggaatcgactaacagaacatcggccccggcgagttgcagggcgcgggctagatgggttgcgatggtcgtcttgcctgacccgcctttctggttaagtacagcgataaccttcatgcgttccccttgcgtatttgtttatttactcatcgcatcatatacgcagcgaccgcatgacgcaagctgttttactcaaatacacatcacctttttagacggcggcgctcggtttcttcagcggccaagctggccggccaggccgccagcttggcatcagacaaaccggccaggatttcatgcagccgcacggttgagacgtgcgcgggcggctcgaacacgtacccggccgcgatcatctccgcctcgatctcttcggtaatgaaaaacggttcgtcctggccgtcctggtgcggtttcatgcttgttcctcttggcgttcattctcggcggccgccagggcgtcggcctcggtcaatgcgtcctcacggaaggcaccgcgccgcctggcctcggtgggcgtcacttcctcgctgcgctcaagtgcgcggtacagggtcgagcgatgcacgccaagcagtgcagccgcctctttcacggtgcggccttcctggtcgatcagctcgcgggcgtgcgcgatctgtgccggggtgagggtagggcgggggccaaacttcacgcctcgggccttggcggcctcgcgcccgctccgggtgcggtcgatgattagggaacgctcgaactcggcaatgccggcgaacacggtcaacaccatgcggccggccggcgtggtggtgtcggcccacggctctgccaggctacgcaggcccgcgccggcctcctggatgcgctcggcaatgtccagtaggtcgcgggtgctgcgggccaggcggtctagcctggtcactgtcacaacgtcgccagggcgtaggtggtcaagcatcctggccagctccgggcggtcgcgcctggtgccggtgatcttctcggaaaacagcttggtgcagccggccgcgtgcagttcggcccgttggttggtcaagtcctggtcgtcggtgctgacgcgggcatagcccagcaggccagcggcggcgctcttgttcatggcgtaatgtctccggttctagtcgcaagtattctactttatgcgactaaaacacgcgacaagaaaacgccaggaaaagggcagggcggcagcctgtcgcgtaacttaggacttgtgcgacatgtcgttttcagaagacggctgcactgaacgtcagaagccgactgcactatagcagcggaggggttggatcaaagtactttgatcccgaggggaaccctgtggttggcatgcacatacaaatggacgaacggataaaccttttcacgcccttttaaatatccgttattctaataaacgctcttttctcttaggtttacccgccaatatatcctgtcaaacactgatagtttaaactgaaggcgggaaacgacaatctgatccaagctcaagctgctctagcattcgccattcaggctgcgcaactgttgggaagggcgatcggtgcgggcctcttcgctattacgccagctggcgaaagggggatgtgctgcaaggcgattaagttgggtaacgccagggttttcccagtcacgacgttgtaaaacgacggccagtgccAAGCTTggcgtgcctgcaggtcaacatggtggagcacgacacacttgtctactccaaaaatatcaaagatacagtctcagaagaccaaagggcaattgagacttttcaacaaagggtaatatccggaaacctcctcggattccattgcccagctatctgtcactttattgtgaagatagtggaaaaggaaggtggctcctacaaatgccatcattgcgataaaggaaaggccatcgttgaagatgcctctgccgacagtggtcccaaagatggacccccacccacgaggagcatcgtggaaaaagaagacgttccaaccacgtcttcaaagcaagtggattgatgtgataacatggtggagcacgacacacttgtctactccaaaaatatcaaagatacagtctcagaagaccaaagggcaattgagacttttcaacaaagggtaatatccggaaacctcctcggattccattgcccagctatctgtcactttattgtgaagatagtggaaaaggaaggtggctcctacaaatgccatcattgcgataaaggaaaggccatcgttgaagatgcctctgccgacagtggtcccaaagatggacccccacccacgaggagcatcgtggaaaaagaagacgttccaaccacgtcttcaaagcaagtggattgatgtgatatctccactgacgtaagggatgacgcacaatcccactatccttcgcaagacccttcctctatataaggaagttcatttcatttggagaggacctcgactctagaggatccccgggtaccgggccccccctcgaggcgcgccaagctatcaaacaagtttgtacaaaaaagcaggctccgaattcgcccttcATGGAGGCCTCTCCGGCTTCAGGTCCTCGCCATCTCATGGACCCCCATATCTTTACCTCCAATTTCAATAACGGCATTGGCAGGCACAAGACATACCTCTGTTATGAGGTCGAGCGGCTCGATAACGGAACCTCAGTGAAGATGGACCAACACAGGGGTTTCCTTCATAACCAAGCGAAAAATCTCCTTTGCGGTTTCTATGGGCGGCACGCGGAGCTGAGATTCTTGGATTTGGTCCCGAGCCTCCAGCTGGATCCCGCGCAAATATACCGCGTCACTTGGTTCATATCATGGAGCCCGTGCTTTAGCTGGGGGTGTGCCGGTGAGGTTCGCGCGTTCCTCCAGGAAAACACCCATGTTCGCCTCCGGATTTTCGCAGCGAGGATTTtCGACTATGATCCACTTTACAAAGAGGCCCTCCAAATGCTTAGAGATGCTGGGGCTCAAGTTTCAATTATGACATACGACGAGTTTAAGCATTGTTGGGACACTTTTGTCGATCATCAGGGTTGTCCCTTCCAGCCGTGGGATGGTCTTGATGAGCATTCGCAAGCTTTGAGTGGCAGGCTTCGCGCGATACTTCAGAACCAGGGTAACtcaggttctgagacccctggcacaagtgagtcagcaacacccgagtccaccatggattacaaggaccacgacggggattacaaggaccacgacattgattacaaggatgatgatgacaagatggctccgaagaagaagaggaaggttggcatccacggggtgccagctgctgacaagaagtactcgatcggcctcgCtattgggactaactctgttggctgggccgtgatcaccgacgagtacaaggtgccctcaaagaagttcaaggtcctgggcaacaccgatcggcattccatcaagaagaatctcattggcgctctcctgttcgacagcggcgagacggctgaggctacgcggctcaagcgcaccgcccgcaggcggtacacgcgcaggaagaatcgcatctgctacctgcaggagattttctccaacgagatggcgaaggttgacgattctttcttccacaggctggaggagtcattcctcgtggaggaggataagaagcacgagcggcatccaatcttcggcaacattgtcgacgaggttgcctaccacgagaagtaccctacgatctaccatctgcggaagaagctcgtggactccacagataaggcggacctccgcctgatctacctcgctctggcccacatgattaagttcaggggccatttcctgatcgagggggatctcaacccggacaatagcgatgttgacaagctgttcatccagctcgtgcagacgtacaaccagctcttcgaggagaaccccattaatgcgtcaggcgtcgacgcgaaggctatcctgtccgctaggctctcgaagtctcggcgcctcgagaacctgatcgcccagctgccgggcgagaagaagaacggcctgttcgggaatctcattgcgctcagcctggggctcacgcccaacttcaagtcgaatttcgatctcgctgaggacgccaagctgcagctctccaaggacacatacgacgatgacctggataacctcctggcccagatcggcgatcagtacgcggacctgttcctcgctgccaagaatctgtcggacgccatcctcctgtctgatattctcagggtgaacaccgagattacgaaggctccgctctcagcctccatgatcaagcgctacgacgagcaccatcaggatctgaccctcctgaaggcgctggtcaggcagcagctccccgagaagtacaaggagatcttcttcgatcagtcgaagaacggctacgctgggtacattgacggcggggcctctcaggaggagttctacaagttcatcaagccgattctggagaagatggacggcacggaggagctgctggtgaagctcaatcgcgaggacctcctgaggaagcagcggacattcgataacggcagcatcccacaccagattcatctcggggagctgcacgctatcctgaggaggcaggaggacttctaccctttcctcaaggataaccgcgagaagatcgagaagattctgactttcaggatcccgtactacgtcggcccactcgctaggggcaactcccgcttcgcttggatgacccgcaagtcagaggagacgatcacgccgtggaacttcgaggaggtggtcgacaagggcgctagcgctcagtcgttcatcgagaggatgacgaatttcgacaagaacctgccaaatgagaaggtgctccctaagcactcgctcctgtacgagtacttcacagtctacaacgagctgactaaggtgaagtatgtgaccgagggcatgaggaagccggctttcctgtctggggagcagaagaaggccatcgtggacctcctgttcaagaccaaccggaaggtcacggttaagcagctcaaggaggactacttcaagaagattgagtgcttcgattcggtcgagatctctggcgttgaggaccgcttcaacgcctccctggggacctaccacgatctcctgaagatcattaaggataaggacttcctggacaacgaggagaatgaggatatcctcgaggacattgtgctgacactcactctgttcgaggaccgggagatgatcgaggagcgcctgaagacttacgcccatctcttcgatgacaaggtcatgaagcagctcaagaggaggaggtacaccggctgggggaggctgagcaggaagctcatcaacggcattcgggacaagcagtccgggaagacgatcctcgacttcctgaagagcgatggcttcgcgaaccgcaatttcatgcagctgattcacgatgacagcctcacattcaaggaggatatccagaaggctcaggtgagcggccagggggactcgctgcacgagcatatcgcgaacctcgctggctcgccagctatcaagaaggggattctgcagaccgtgaaggttgtggacgagctggtgaaggtcatgggcaggcacaagcctgagaacatcgtcattgagatggcccgggagaatcagaccacgcagaagggccagaagaactcacgcgagaggatgaagaggatcgaggagggcattaaggagctggggtcccagatcctcaaggagcacccggtggagaacacgcagctgcagaatgagaagctctacctgtactacctccagaatggccgcgatatgtatgtggaccaggagctggatattaacaggctcagcgattacgacgtcgatcatatcgttccacagtcattcctgaaggatgactccattgacaacaaggtcctcaccaggtcggacaagaaccggggcaagtctgataatgttccttcagaggaggtcgttaagaagatgaagaactactggcgccagctcctgaatgccaagctgatcacgcagcggaagttcgataacctcacaaaggctgagaggggcgggctctctgagctggacaaggcgggcttcatcaagaggcagctggtcgagacacggcagatcactaagcacgttgcgcagattctcgactcacggatgaacactaagtacgatgagaatgacaagctgatccgcgaggtgaaggtcatcaccctgaagtcaaagctcgtctccgacttcaggaaggatttccagttctacaaggttcgggagatcaacaattaccaccatgcccatgacgcgtacctgaacgcggtggtcggcacagctctgatcaagaagtacccaaagctcgagagcgagttcgtgtacggggactacaaggtttacgatgtgaggaagatgatcgccaagtcggagcaggagattggcaaggctaccgccaagtacttcttctactctaacattatgaatttcttcaagacagagatcactctggccaatggcgagatccggaagcgccccctcatcgagacgaacggcgagacgggggagatcgtgtgggacaagggcagggatttcgcgaccgtcaggaaggttctctccatgccacaagtgaatatcgtcaagaagacagaggtccagactggcgggttctctaaggagtcaattctgcctaagcggaacagcgacaagctcatcgcccgcaagaaggactgggatccgaagaagtacggcgggttcgacagccccactgtggcctactcggtcctggttgtggcgaaggttgagaagggcaagtccaagaagctcaagagcgtgaaggagctgctggggatcacgattatggagcgctccagcttcgagaagaacccgatcgatttcctggaggcgaagggctacaaggaggtgaagaaggacctgatcattaagctccccaagtactcactcttcgagctggagaacggcaggaagcggatgctggcttccgctggcgagctgcagaaggggaacgagctggctctgccgtccaagtatgtgaacttcctctacctggcctcccactacgagaagctcaagggcagccccgaggacaacgagcagaagcagctgttcgtcgagcagcacaagcattacctcgacgagatcattgagcagatttccgagttctccaagcgcgtgatcctggccgacgcgaatctggataaggtcctctccgcgtacaacaagcaccgcgacaagccaatcagggagcaggctgagaatatcattcatctcttcaccctgacgaacctcggcgcccctgctgctttcaagtacttcgacacaactatcgatcgcaagaggtacacaagcactaaggaggtcctggacgcgaccctcatccaccagtcgattaccggcctctacgagacgcgcatcgacctgtctcagctcgggggcgacaagcggccagcggcgacgaagaaggcggggcaggcgaagaagaagaagggagacggatcaggcgggtcaacaaacctcagtgacatcatagagaaggaaactggtaagcaactggttattcaagagagcattctgatgctccctgaggaagtcgaggaagttataggaaacaagcctgagagcgatatactcgtgcatacggcgtatgacgagagcacggatgaaaatgtgatgctcttgaccagcgatgcgccagaatataaaccctgggcactggttattcaggactctaacggggaaaataaaatcaagatgctctcaggtggctccccaaagaagaaacgcaaggtttagATTATCAGAGTGGTGGTGGGCCCATAACCCACAGGTCCGCTCTGATgacgtccgatcgttcaaacatttggcaataaagtttcttaagattgaatcctgttgccggtcttgcgatgattatcatataatttctgttgaattacgttaagcatgtaataattaacatgtaatgcatgacgttatttatgagatgggtttttatgattagagtcccgcaattatacatttaatacgcgatagaaaacaaaatatagcgcgcaaactaggataaattatcgcgcgcggtgtcatctatgttactagatcgggaattgatcccccctcgacagcttccggaaagggcgaattcgcaactttgtatacaaaagttgccccatggcgttccctctagataacgcaggatccccaagtggtggctattcgacgagtcagtaataaacggcgtcaaagtggttgcagccggcacacacgagtcgtgtttatcaactcaaagcacaaatacttttcctcaacctaaaaataaggcaattagccaaaaacaactttgcgtgtaaacaacgctcaatacacgtgtcattttattattagctattgcttcaccgccttagctttctcgtgacctagtcgtcctcgtcttttcttcttcttcttctataaaacaatacccaaagagctcttcttcttcacaattcagatttcaatttctcaaaatcttaaaaactttctctcaattctctctaccgtgatcaaggtaaatttctgtgttccttattctctcaaaatcttcgattttgttttcgttcgatcccaatttcgtatatgttctttggtttagattctgttaatcttagatcgaagacgattttctgggtttgatcgttagatatcatcttaattctcgattagggtttcatagatatcatccgatttgttcaaataatttgagttttgtcgaataattactcttcgatttgtgatttctatGtagatctggtgttagtttctagtttgtgcgatcgaatttgtcgattaatctgagtttttctgattaacagAATGGTGACAGGGGGAATGGCAAGCAAGTGGGATCAGAAGGGTATGGACATTGCCTATGAGGAGGCGGCCTTAGGTTACAAAGAGGGTGGTGTTCCTATTGGCGGATGTCTTATCAATAACAAAGACGGAAGTGTTCTCGGTCGTGGTCACAACATGAGATTTCAAAAGGGATCCGCCACACTACATGGTGAGATCTCCACTTTGGAAAACTGTGGGAGATTAGAGGGCAAAGTGTACAAAGATACCACTTTGTATACGACGCTGTCTCCATGCGACATGTGTACAGGTGCCATCATCATGTATGGTATTCCACGCTGTGTTGTCGGTGAGAACGTTAATTTCAAAAGTAAGGGCGAGAAATATTTACAAACTAGAGGTCACGAGGTTGTTGTTGTTGACGATGAGAGGTGTAAAAAGATCATGAAACAATTTATCGATGAAAGACCTCAGGATTGGTTTGAAGATATTGGTGAGgctTCCGccAAGATCGTGGAAGTCAAACACCCACTCGTCAAACACAAGCTGGGACTGATGCGTGAGCAAGATATCAGCACCAAGCGCTTTCGCGAACTCGCTTCCGAAGTGGGTAGCCTGCTGACTTACGAAGCGACCGCCGACCTCGAAACGGAAAAAGTAACTATCGAAGGCTGGAACGGCCCGGTAGAAATCGACCAGATCAAAGGTAAGAAAATTACCGTTGTGCCAATTCTGCGTGCGGGTCTTGGTATGATGGACGGTGTGCTGGAAAACGTTCCGAGCGCGCGCATCAGCGTTGTCGGTATGTACCGTAATGAAGAAACGCTGGAGCCGGTACCGTACTTCCAGAAACTGGTTTCTAACATCGATGAGCGTATGGCGCTGATCGTTGACCCAATGCTGGCAACCGGTGGTTCCGTTATCGCGACCATCGACCTGCTGAAAAAAGCGGGCTGCAGCAGCATCAAAGTTCTGGTGCTGGTAGCTGCGCCAGAAGGTATCGCTGCGCTGGAAAAAGCGCACCCGGACGTCGAACTGTATACCGCATCGATTGATCAGGGACTGAACGAGCACGGATACATTATTCCGGGCCTCGGCGATGCCGGTGACAAAATCTTTGGTACGAAAtaGccgatcgttcaaacatttggcaataaagtttcttaagattgaatcctgttgccggtcttgcgatgattatcatataatttctgttgaattacgttaagcatgtaataattaacatgtaatgcatgacgttatttatgagatgggtttttatgattagagtcccgcaattatacatttaatacgcgatagaaaacaaaatatagcgcgcaaactaggataaattatcgcgcgcggtgtcatctatgttactagatcgggaattgatcccccctcgacagcatgTTTACTTTAAATTTTTCTTATGGCTCAGCCTGTGATGGATAACTGAATCAAACAAATGGCGTCTGGGTTTAAGAAcATCTGTTTTGGCTATGTTGGACGAAACAAGTGAACTTTTAGGATCAACTTCAGTTTATATACGGAGCTTATATCGAGCAATAAGATAAGTGGGCTTTTTATGTAATTTAATGGGCTATCGTCCATATATTCACTAATACCCATGCCCAGTACCCATGTATGCGTTTCATATAAGCTCCTAATTTCTCCCACATCGCTCAAATCTAAACAAATCTTGTTGTATATATAACACTGAGGGAGCACCATTGGTCACAGGTCCCTCGGAGGATGATGTTTTAGAGCTAGAAATAGCAAGTTAAAATAAGGCTAGTCCGTTATCAACTTGAAAAAGTGGCACCGAGTCGGTGCATTATCAGAGTGGTGGTGGGCCCATAACCCACAGGTCCGCTCTGATTCCCTTTCCTTTTTTCTTTTTTTTGCCATAAACTTAAATTTGTATATCGATCATTGTAGATATTGAAAACCTAGAACAAACCAACATCCATGTGAATGTCTTTCATGACTGATTTAGAGATAATTCTTGAATTTTGGAACTAGAATCTATAATGAGCCTaaattaaaacattgtgggacTTTACTTTAAATTTTTCTTATGGCTCAGCCTGTGATGGATAACTGAATCAAACAAATGGCGTCTGGGTTTAAGAAcATCTGTTTTGGCTATGTTGGACGAAACAAGTGAACTTTTAGGATCAACTTCCGTTTATATACGGAGCTTATATCGAGCAATAAGATAAGTGGGCTTTTTATGTAATTTAATGGGCTATCGTCCATATATTCACTAATACCCATGCCCAGTACCCATGTATGCGTTTCATATAAGCTCCTAATTTCTCCCACATCGCTCAAATCTAAACAAATCTTGTTGTATATATAACACTGAGGGAGCACCATTGGTCACAGGTCCCGCGGAGGATGATGTTTTAGAGCTAGAAATAGCAAGTTAAAATAAGGCTAGTCCGTTATCAACTTGAAAAAGTGGCACCGAGTCGGTGCATTATCAGAGTGGTGGTGGGCCCATAACCCACAGGTCCGCTCTGATTCCCTTTCCTTTTTTCTTTTTTTTGCCATAAACTTAAATTTGTATATCGATCATTGTAGATATTGAAAACCTAGAACAAACCAACATCCATGTGAATGTCTTTCATGACTGATTTAGAGATAATTCTTGAATTTTGGAACTAGAATCTATAATGAGCCTaaattaaaacattgtgccagTTTACTTTAAATTTTTCTTATGGCTCAGCCTGTGATGGATAACTGAATCAAACAAATGGCGTCTGGGTTTAAGAAcATCTGTTTTGGCTATGTTGGACGAAACAAGTGAACTTTTAGGATCAACTTCCGTTTATATACGGAGCTTATATCGAGCAATAAGATAAGTGGGCTTTTTATGTAATTTAATGGGCTATCGTCCATATATTCACTAATACCCATGCCCAGTACCCATGTATGCGTTTCATATAAGCTCCTAATTTCTCCCACATCGCTCAAATCTAAACAAATCTTGTTGTATATATAACACTGAGGGAGCACCATTGGTCATCATCGATGTATTTTGGCTGCAGTTTTAGAGCTAGAAATAGCAAGTTAAAATAAGGCTAGTCCGTTATCAACTTGAAAAAGTGGCACCGAGTCGGTGCATTATCAGAGTGGTGGTGGGCCCATAACCCACAGGTCCGCTCTGATTCCCTTTCCTTTTTTCTTTTTTTTGCCATAAACTTAAATTTGTATATCGATCATTGTAGATATTGAAAACCTAGAACAAACCAACATCCATGTGAATGTCTTTCATGACTGATTTAGAGATAATTCTTGAATTTTGGAACTAGAATCTATAATGAGCCTaaattaaaacattgtgTGTTactagtaagggcgaattcgacccagctttcttgtacaaagtggttcgataattcttaattaactagttctagagcggccgccaccgcggtggagctcgaatttccccgatcgttcaaacatttggcaataaagtttcttaagattgaatcctgttgccggtcttgcgatgattatcatataatttctgttgaattacgttaagcatgtaataattaacatgtaatgcatgacgttatttatgagatgggtttttatgattagagtcccgcaattatacatttaatacgcgatagaaaacaaaatatagcgcgcaaactaggataaattatcgcgcgcggtgtcatctatgttactgaattcgtaatcatggtcatagAAGCTTgcatgcctgcaggtcgactctagaggatccccgggtaccgagctcgaattcgtaatcatgtcatagctgtttcctgtgtgaaattgttatccgctcacaattccacacaacatacgagccggaagcataaagtgtaaagcctggggtgcctaatgagtgagctaactcacattaattgcgttgcgctcactgcccgctttccagtcgggaaacctgtcgtgccagctgcattaatgaatcggccaacgcgcggggagaggcggtttgcgtattggctagagcagcttgccaacatggtggagcacgacactctcgtctactccaagaatatcaaagatacagtctcagaagaccaaagggctattgagacttttcaacaaagggtaatatcgggaaacctcctcggattccattgcccagctatctgtcacttcatcaaaaggacagtagaaaaggaaggtggcacctacaaatgccatcattgcgataaaggaaaggctatcgttcaagatgcctctgccgacagtggtcccaaagatggacccccacccacgaggagcatcgtggaaaaagaagacgttccaaccacgtcttcaaagcaagtggattgatgtgataacatggtggagcacgacactctcgtctactccaagaatatcaaagatacagtctcagaagaccaaagggctattgagacttttcaacaaagggtaatatcgggaaacctcctcggattccattgcccagctatctgtcacttcatcaaaaggacagtagaaaaggaaggtggcacctacaaatgccatcattgcgataaaggaaaggctatcgttcaagatgcctctgccgacagtggtcccaaagatggacccccacccacgaggagcatcgtggaaaaagaagacgttccaaccacgtcttcaaagcaagtggattgatgtgatatctccactgacgtaagggatgacgcacaatcccactatccttcgcaagaccttcctctatataaggaagttcatttcatttggagaggacacgctgaaatcaccagtctctctctacaaatctatctctctcgagctttcgcagatctgtcgatcgaccatggggattgaacaagatggattgcacgcaggttctccggccgcttgggtggagaggctattcggctatgactgggcacaacagacaatcggctgctctgatgccgccgtgttccggctgtcagcgcaggggcgcccggttctttttgtcaagaccgacctgtccggtgccctgaatgaactccaggacgaggcagcgcggctatcgtggctggccacgacgggcgttccttgcgcagctgtgctcgacgttgtcactgaagcgggaagggactggctgctattgggcgaagtgccggggcaggatctcctgtcatctcaccttgctcctgccgagaaagtatccatcatggctgatgcaatgcggcggctgcatacgcttgatccggctacctgcccattcgaccaccaagcgaaacatcgcatcgagcgagcacgtactcggatggaagccggtcttgtcgatcaggatgatctggacgaagagcatcaggggctcgcgccagccgaactgttcgccaggctcaaggcgcgcatgcccgacggcgaggatctcgtcgtgacacatggcgatgcctgcttgccgaatatcatggtggaaaatggccgcttttctggattcatcgactgtggccggctgggtgtggcggaccgctatcaggacatagcgttggctacccgtgatattgctgaagagcttggcggcgaatgggctgaccgcttcctcgtgctttacggtatcgccgctcccgattcgcagcgcatcgccttctatcgccttcttgacgagttcttctgagcgggactctggggttcggatcgatcctctagctagagtcgatcgacaagctcgagtttctccataataatgtgtgagtagttcccagataagggaattagggttcctatagggtttcgctcatgtgttgagcatataagaaacccttagtatgtatttgtatttgtaaaatacttctatcaataaaatttctaattcctaaaaccaaaatccagtactaaaatccagatcccccgaattaattcggcgttaattcagtacattaaaaacgtccgcaatgtgttattaagttgtctaagcgtcaatttgtttacaccacaatatatcctgcca

**Supplemental data 3.** Complete sequence of the poplar construct without TLS.

**Vector components legend:**

- Base editing system: AtUBQ10 promoter, ha3A, Cas9, UGI, NOS terminator.
- Toxin genes: AtUBQ10 promoter, ScFCY, UPP, NOS terminator.
- sgRNA: AtU3 promoter, PtALS_gRNA, sgRNA scaffold, AtU3 terminator, AtU3 promoter, Pt4CL1_sgRNA, sgRNA scaffold, AtU3 terminator.
- The right and left T-DNA borders are underlined.

**pLR5478 (**12,548 bp**)**

gtttacccgccaatatatcctgtcaaacactgatagtttaaactgaaggcgggaaacgacaatctgatccaagctcaagctgctctagcattcgccattcaggctgcgcaactgttgggaagggcgatcggtgcgggcctcttcgctattacgccagctggcgaaagggggatgtgctgcaaggcgattaagttgggtaacgccagggttttcccagtcacgacgttgtaaaacgacggccagtgccaagctttcgacgagtcagtaataaacggcgtcaaagtggttgcagccggcacacacgagtcgtgtttatcaactcaaagcacaaatacttttcctcaacctaaaaataaggcaattagccaaaaacaactttgcgtgtaaacaacgctcaatacacgtgtcattttattattagctattgcttcaccgccttagctttctcgtgacctagtcgtcctcgtcttttcttcttcttcttctataaaacaatacccaaagagctcttcttcttcacaattcagatttcaatttctcaaaatcttaaaaactttctctcaattctctctaccgtgatcaaggtaaatttctgtgttccttattctctcaaaatcttcgattttgttttcgttcgatcccaatttcgtatatgttctttggtttagattctgttaatcttagatcgaagacgattttctgggtttgatcgttagatatcatcttaattctcgattagggtttcatagatatcatccgatttgttcaaataatttgagttttgtcgaataattactcttcgatttgtgatttctatctagatctggtgttagtttctagtttgtgcgatcgaatttgtagattaatctgagtttttctgattaacagggtaccgggccccccctcgaggcgcgccaagctatcaaacaagtttgtacaaaaaagcaggctccgaattcgcccttcATGGAGGCCTCTCCGGCTTCAGGTCCTCGCCATCTCATGGACCCCCATATCTTTACCTCCAATTTCAATAACGGCATTGGCAGGCACAAGACATACCTCTGTTATGAGGTCGAGCGGCTCGATAACGGAACCTCAGTGAAGATGGACCAACACAGGGGTTTCCTTCATAACCAAGCGAAAAATCTCCTTTGCGGTTTCTATGGGCGGCACGCGGAGCTGAGATTCTTGGATTTGGTCCCGAGCCTCCAGCTGGATCCCGCGCAAATATACCGCGTCACTTGGTTCATATCATGGAGCCCGTGCTTTAGCTGGGGGTGTGCCGGTGAGGTTCGCGCGTTCCTCCAGGAAAACACCCATGTTCGCCTCCGGATTTTCGCAGCGAGGATTTtCGACTATGATCCACTTTACAAAGAGGCCCTCCAAATGCTTAGAGATGCTGGGGCTCAAGTTTCAATTATGACATACGACGAGTTTAAGCATTGTTGGGACACTTTTGTCGATCATCAGGGTTGTCCCTTCCAGCCGTGGGATGGTCTTGATGAGCATTCGCAAGCTTTGAGTGGCAGGCTTCGCGCGATACTTCAGAACCAGGGTAACtcaggttctgagacccctggcacaagtgagtcagcaacacccgagtccaccatggattacaaggaccacgacggggattacaaggaccacgacattgattacaaggatgatgatgacaagatggctccgaagaagaagaggaaggttggcatccacggggtgccagctgctgacaagaagtactcgatcggcctcgCtattgggactaactctgttggctgggccgtgatcaccgacgagtacaaggtgccctcaaagaagttcaaggtcctgggcaacaccgatcggcattccatcaagaagaatctcattggcgctctcctgttcgacagcggcgagacggctgaggctacgcggctcaagcgcaccgcccgcaggcggtacacgcgcaggaagaatcgcatctgctacctgcaggagattttctccaacgagatggcgaaggttgacgattctttcttccacaggctggaggagtcattcctcgtggaggaggataagaagcacgagcggcatccaatcttcggcaacattgtcgacgaggttgcctaccacgagaagtaccctacgatctaccatctgcggaagaagctcgtggactccacagataaggcggacctccgcctgatctacctcgctctggcccacatgattaagttcaggggccatttcctgatcgagggggatctcaacccggacaatagcgatgttgacaagctgttcatccagctcgtgcagacgtacaaccagctcttcgaggagaaccccattaatgcgtcaggcgtcgacgcgaaggctatcctgtccgctaggctctcgaagtctcggcgcctcgagaacctgatcgcccagctgccgggcgagaagaagaacggcctgttcgggaatctcattgcgctcagcctggggctcacgcccaacttcaagtcgaatttcgatctcgctgaggacgccaagctgcagctctccaaggacacatacgacgatgacctggataacctcctggcccagatcggcgatcagtacgcggacctgttcctcgctgccaagaatctgtcggacgccatcctcctgtctgatattctcagggtgaacaccgagattacgaaggctccgctctcagcctccatgatcaagcgctacgacgagcaccatcaggatctgaccctcctgaaggcgctggtcaggcagcagctccccgagaagtacaaggagatcttcttcgatcagtcgaagaacggctacgctgggtacattgacggcggggcctctcaggaggagttctacaagttcatcaagccgattctggagaagatggacggcacggaggagctgctggtgaagctcaatcgcgaggacctcctgaggaagcagcggacattcgataacggcagcatcccacaccagattcatctcggggagctgcacgctatcctgaggaggcaggaggacttctaccctttcctcaaggataaccgcgagaagatcgagaagattctgactttcaggatcccgtactacgtcggcccactcgctaggggcaactcccgcttcgcttggatgacccgcaagtcagaggagacgatcacgccgtggaacttcgaggaggtggtcgacaagggcgctagcgctcagtcgttcatcgagaggatgacgaatttcgacaagaacctgccaaatgagaaggtgctccctaagcactcgctcctgtacgagtacttcacagtctacaacgagctgactaaggtgaagtatgtgaccgagggcatgaggaagccggctttcctgtctggggagcagaagaaggccatcgtggacctcctgttcaagaccaaccggaaggtcacggttaagcagctcaaggaggactacttcaagaagattgagtgcttcgattcggtcgagatctctggcgttgaggaccgcttcaacgcctccctggggacctaccacgatctcctgaagatcattaaggataaggacttcctggacaacgaggagaatgaggatatcctcgaggacattgtgctgacactcactctgttcgaggaccgggagatgatcgaggagcgcctgaagacttacgcccatctcttcgatgacaaggtcatgaagcagctcaagaggaggaggtacaccggctgggggaggctgagcaggaagctcatcaacggcattcgggacaagcagtccgggaagacgatcctcgacttcctgaagagcgatggcttcgcgaaccgcaatttcatgcagctgattcacgatgacagcctcacattcaaggaggatatccagaaggctcaggtgagcggccagggggactcgctgcacgagcatatcgcgaacctcgctggctcgccagctatcaagaaggggattctgcagaccgtgaaggttgtggacgagctggtgaaggtcatgggcaggcacaagcctgagaacatcgtcattgagatggcccgggagaatcagaccacgcagaagggccagaagaactcacgcgagaggatgaagaggatcgaggagggcattaaggagctggggtcccagatcctcaaggagcacccggtggagaacacgcagctgcagaatgagaagctctacctgtactacctccagaatggccgcgatatgtatgtggaccaggagctggatattaacaggctcagcgattacgacgtcgatcatatcgttccacagtcattcctgaaggatgactccattgacaacaaggtcctcaccaggtcggacaagaaccggggcaagtctgataatgttccttcagaggaggtcgttaagaagatgaagaactactggcgccagctcctgaatgccaagctgatcacgcagcggaagttcgataacctcacaaaggctgagaggggcgggctctctgagctggacaaggcgggcttcatcaagaggcagctggtcgagacacggcagatcactaagcacgttgcgcagattctcgactcacggatgaacactaagtacgatgagaatgacaagctgatccgcgaggtgaaggtcatcaccctgaagtcaaagctcgtctccgacttcaggaaggatttccagttctacaaggttcgggagatcaacaattaccaccatgcccatgacgcgtacctgaacgcggtggtcggcacagctctgatcaagaagtacccaaagctcgagagcgagttcgtgtacggggactacaaggtttacgatgtgaggaagatgatcgccaagtcggagcaggagattggcaaggctaccgccaagtacttcttctactctaacattatgaatttcttcaagacagagatcactctggccaatggcgagatccggaagcgccccctcatcgagacgaacggcgagacgggggagatcgtgtgggacaagggcagggatttcgcgaccgtcaggaaggttctctccatgccacaagtgaatatcgtcaagaagacagaggtccagactggcgggttctctaaggagtcaattctgcctaagcggaacagcgacaagctcatcgcccgcaagaaggactgggatccgaagaagtacggcgggttcgacagccccactgtggcctactcggtcctggttgtggcgaaggttgagaagggcaagtccaagaagctcaagagcgtgaaggagctgctggggatcacgattatggagcgctccagcttcgagaagaacccgatcgatttcctggaggcgaagggctacaaggaggtgaagaaggacctgatcattaagctccccaagtactcactcttcgagctggagaacggcaggaagcggatgctggcttccgctggcgagctgcagaaggggaacgagctggctctgccgtccaagtatgtgaacttcctctacctggcctcccactacgagaagctcaagggcagccccgaggacaacgagcagaagcagctgttcgtcgagcagcacaagcattacctcgacgagatcattgagcagatttccgagttctccaagcgcgtgatcctggccgacgcgaatctggataaggtcctctccgcgtacaacaagcaccgcgacaagccaatcagggagcaggctgagaatatcattcatctcttcaccctgacgaacctcggcgcccctgctgctttcaagtacttcgacacaactatcgatcgcaagaggtacacaagcactaaggaggtcctggacgcgaccctcatccaccagtcgattaccggcctctacgagacgcgcatcgacctgtctcagctcgggggcgacaagcggccagcggcgacgaagaaggcggggcaggcgaagaagaagaagggagacggatcaggcgggtcaacaaacctcagtgacatcatagagaaggaaactggtaagcaactggttattcaagagagcattctgatgctccctgaggaagtcgaggaagttataggaaacaagcctgagagcgatatactcgtgcatacggcgtatgacgagagcacggatgaaaatgtgatgctcttgaccagcgatgcgccagaatataaaccctgggcactggttattcaggactctaacggggaaaataaaatcaagatgctctcaggtggctccccaaagaagaaacgcaaggtttagtggcggccgccgacgtccgatcgttcaaacatttggcaataaagtttcttaagattgaatcctgttgccggtcttgcgatgattatcatataatttctgttgaattacgttaagcatgtaataattaacatgtaatgcatgacgttatttatgagatgggtttttatgattagagtcccgcaattatacatttaatacgcgatagaaaacaaaatatagcgcgcaaactaggataaattatcgcgcgcggtgtcatctatgttactagatcgggaattgatcccccctcgacagcttccggaaagggcgaattcgcaactttgtatacaaaagttgccccatggcgttccctctagataacgcaggatccccaagtggtggctattcgacgagtcagtaataaacggcgtcaaagtggttgcagccggcacacacgagtcgtgtttatcaactcaaagcacaaatacttttcctcaacctaaaaataaggcaattagccaaaaacaactttgcgtgtaaacaacgctcaatacacgtgtcattttattattagctattgcttcaccgccttagctttctcgtgacctagtcgtcctcgtcttttcttcttcttcttctataaaacaatacccaaagagctcttcttcttcacaattcagatttcaatttctcaaaatcttaaaaactttctctcaattctctctaccgtgatcaaggtaaatttctgtgttccttattctctcaaaatcttcgattttgttttcgttcgatcccaatttcgtatatgttctttggtttagattctgttaatcttagatcgaagacgattttctgggtttgatcgttagatatcatcttaattctcgattagggtttcatagatatcatccgatttgttcaaataatttgagttttgtcgaataattactcttcgatttgtgatttctatGtagatctggtgttagtttctagtttgtgcgatcgaatttgtcgattaatctgagtttttctgattaacagAATGGTGACAGGGGGAATGGCAAGCAAGTGGGATCAGAAGGGTATGGACATTGCCTATGAGGAGGCGGCCTTAGGTTACAAAGAGGGTGGTGTTCCTATTGGCGGATGTCTTATCAATAACAAAGACGGAAGTGTTCTCGGTCGTGGTCACAACATGAGATTTCAAAAGGGATCCGCCACACTACATGGTGAGATCTCCACTTTGGAAAACTGTGGGAGATTAGAGGGCAAAGTGTACAAAGATACCACTTTGTATACGACGCTGTCTCCATGCGACATGTGTACAGGTGCCATCATCATGTATGGTATTCCACGCTGTGTTGTCGGTGAGAACGTTAATTTCAAAAGTAAGGGCGAGAAATATTTACAAACTAGAGGTCACGAGGTTGTTGTTGTTGACGATGAGAGGTGTAAAAAGATCATGAAACAATTTATCGATGAAAGACCTCAGGATTGGTTTGAAGATATTGGTGAGgctTCCGccAAGATCGTGGAAGTCAAACACCCACTCGTCAAACACAAGCTGGGACTGATGCGTGAGCAAGATATCAGCACCAAGCGCTTTCGCGAACTCGCTTCCGAAGTGGGTAGCCTGCTGACTTACGAAGCGACCGCCGACCTCGAAACGGAAAAAGTAACTATCGAAGGCTGGAACGGCCCGGTAGAAATCGACCAGATCAAAGGTAAGAAAATTACCGTTGTGCCAATTCTGCGTGCGGGTCTTGGTATGATGGACGGTGTGCTGGAAAACGTTCCGAGCGCGCGCATCAGCGTTGTCGGTATGTACCGTAATGAAGAAACGCTGGAGCCGGTACCGTACTTCCAGAAACTGGTTTCTAACATCGATGAGCGTATGGCGCTGATCGTTGACCCAATGCTGGCAACCGGTGGTTCCGTTATCGCGACCATCGACCTGCTGAAAAAAGCGGGCTGCAGCAGCATCAAAGTTCTGGTGCTGGTAGCTGCGCCAGAAGGTATCGCTGCGCTGGAAAAAGCGCACCCGGACGTCGAACTGTATACCGCATCGATTGATCAGGGACTGAACGAGCACGGATACATTATTCCGGGCCTCGGCGATGCCGGTGACAAAATCTTTGGTACGAAAtaGccgatcgttcaaacatttggcaataaagtttcttaagattgaatcctgttgccggtcttgcgatgattatcatataatttctgttgaattacgttaagcatgtaataattaacatgtaatgcatgacgttatttatgagatgggtttttatgattagagtcccgcaattatacatttaatacgcgatagaaaacaaaatatagcgcgcaaactaggataaattatcgcgcgcggtgtcatctatgttactagatcgggaattgatcccccctcgacagcatgTTTACTTTAAATTTTTCTTATGGCTCAGCCTGTGATGGATAACTGAATCAAACAAATGGCGTCTGGGTTTAAGAAcATCTGTTTTGGCTATGTTGGACGAAACAAGTGAACTTTTAGGATCAACTTCAGTTTATATACGGAGCTTATATCGAGCAATAAGATAAGTGGGCTTTTTATGTAATTTAATGGGCTATCGTCCATATATTCACTAATACCCATGCCCAGTACCCATGTATGCGTTTCATATAAGCTCCTAATTTCTCCCACATCGCTCAAATCTAAACAAATCTTGTTGTATATATAACACTGAGGGAGCACCATTGGTCACAGGTTCCGCGGCGAATGATGTTTTAGAGCTAGAAATAGCAAGTTAAAATAAGGCTAGTCCGTTATCAACTTGAAAAAGTGGCACCGAGTCGGTGCTTTTTTTTCCCTTTCCTTTTTTCTTTTTTTTGCCATAAACTTAAATTTGTATATCGATCATTGTAGATATTGAAAACCTAGAACAAACCAACATCCATGTGAATGTCTTTCATGACTGATTTAGAGATAATTCTTGAATTTTGGAACTAGAATCTATAATGAGCCTaaattaaaacattgtgggacTTTACTTTAAATTTTTCTTATGGCTCAGCCTGTGATGGATAACTGAATCAAACAAATGGCGTCTGGGTTTAAGAAcATCTGTTTTGGCTATGTTGGACGAAACAAGTGAACTTTTAGGATCAACTTCCGTTTATATACGGAGCTTATATCGAGCAATAAGATAAGTGGGCTTTTTATGTAATTTAATGGGCTATCGTCCATATATTCACTAATACCCATGCCCAGTACCCATGTATGCGTTTCATATAAGCTCCTAATTTCTCCCACATCGCTCAAATCTAAACAAATCTTGTTGTATATATAACACTGAGGGAGCACCATTGGTCAAGTGTGGCTCAACAGGTAGA GTTTTAGAGCTAGAAATAGCAAGTTAAAATAAGGCTAGTCCGTTATCAACTTGAAAAAGTGGCACCGAGTCGGTGCTTTTTTTTCCCTTTCCTTTTTTCTTTTTTTTGCCATAAACTTAAATTTGTATATCGATCATTGTAGATATTGAAAACCTAGAACAAACCAACATCCATGTGAATGTCTTTCATGACTGATTTAGAGATAATTCTTGAATTTTGGAACTAGAATCTATAATGAGCCTaaattaaaacattgtgCCAGactagtaagggcAaattcgacccagctttcttgtacaaagtggttcgataattcttaattaactagttctagagcggccgccaccgcggtggagctcgaatttccccgatcgttcaaacatttggcaataaagtttcttaagattgaatcctgttgccggtcttgcgatgattatcatataatttctgttgaattacgttaagcatgtaataattaacatgtaatgcatgacgttatttatgagatgggtttttatgattagagtcccgcaattatacatttaatacgcgatagaaaacaaaatatagcgcgcaaactaggataaattatcgcgcgcggtgtcatctatgttactagatcgggaattcgtaatcatgtcatagctgtttcctgtgtgaaattgttatccgctcacaattccacacaacatacgagccggaagcataaagtgtaaagcctggggtgcctaatgagtgagctaactcacattaattgcgttgcgctcactgcccgctttccagtcgggaaacctgtcgtgccagctgcattaatgaatcggccaacgcgcggggagaggcggtttgcgtattggctagagcagcttgccaacatggtggagcacgacactctcgtctactccaagaatatcaaagatacagtctcagaagaccaaagggctattgagacttttcaacaaagggtaatatcgggaaacctcctcggattccattgcccagctatctgtcacttcatcaaaaggacagtagaaaaggaaggtggcacctacaaatgccatcattgcgataaaggaaaggctatcgttcaagatgcctctgccgacagtggtcccaaagatggacccccacccacgaggagcatcgtggaaaaagaagacgttccaaccacgtcttcaaagcaagtggattgatgtgaacatggtggagcacgacactctcgtctactccaagaatatcaaagatacagtctcagaagaccaaagggctattgagacttttcaacaaagggtaatatcgggaaacctcctcggattccattgcccagctatctgtcacttcatcaaaaggacagtagaaaaggaaggtggcacctacaaatgccatcattgcgataaaggaaaggctatcgttcaagatgcctctgccgacagtggtcccaaagatggacccccacccacgaggagcatcgtggaaaaagaagacgttccaaccacgtcttcaaagcaagtggattgatgtgatatctccactgacgtaagggatgacgcacaatcccactatccttcgcaagacccttcctctatataaggaagttcatttcatttggagaggacacgctgaaatcaccagtctctctctacaaatctatctctctcgagctttcgcagatccggggggcaatgagatatgaaaaagcctgaactcaccgcgacgtctgtcgagaagtttctgatcgaaaagttcgacagcgtctccgacctgatgcagctctcggagggcgaagaatctcgtgctttcagcttcgatgtaggagggcgtggatatgtcctgcgggtaaatagctgcgccgatggtttctacaaagatcgttatgtttatcggcactttgcatcggccgcgctcccgattccggaagtgcttgacattggggagtttagcgagagcctgacctattgcatctcccgccgttcacagggtgtcacgttgcaagacctgcctgaaaccgaactgcccgctgttctacaaccggtcgcggaggctatggatgcgatcgctgcggccgatcttagccagacgagcgggttcggcccattcggaccgcaaggaatcggtcaatacactacatggcgtgatttcatatgcgcgattgctgatccccatgtgtatcactggcaaactgtgatggacgacaccgtcagtgcgtccgtcgcgcaggctctcgatgagctgatgctttgggccgaggactgccccgaagtccggcacctcgtgcacgcggatttcggctccaacaatgtcctgacggacaatggccgcataacagcggtcattgactggagcgaggcgatgttcggggattcccaatacgaggtcgccaacatcttcttctggaggccgtggttggcttgtatggagcagcagacgcgctacttcgagcggaggcatccggagcttgcaggatcgccacgactccgggcgtatatgctccgcattggtcttgaccaactctatcagagcttggttgacggcaatttcgatgatgcagcttgggcgcagggtcgatgcgacgcaatcgtccgatccggagccgggactgtcgggcgtacacaaatcgcccgcagaagcgcggccgtctggaccgatggctgtgtagaagtactcgccgatagtggaaaccgacgccccagcactcgtccgagggcaaagaaatagagtagatgccgaccgggatctgtcgatcgacaagctcgagtttctccataataatgtgtgagtagttcccagataagggaattagggttcctatagggtttcgctcatgtgttgagcatataagaaacccttagtatgtatttgtatttgtaaaatacttctatcaataaaatttctaattcctaaaaccaaaatccagtactaaaatccagatcccccgaattaattcggcgttaattcagtacattaaaaacgtccgcaatgtgttattaagttgtctaagcgtcaatttgtttacaccacaatatatcctgcca

**Supplemental data 4.** Complete sequence of the poplar construct with TLS.

**Vector components legend:**

- Base editing system: AtUBQ10 promoter, ha3A, Cas9, UGI, TLS sequence, NOS terminator.
- Toxin genes: AtUBQ10 promoter, ScFCY, UPP, NOS terminator.
- sgRNA: AtU3 promoter, PtALS_gRNA, sgRNA scaffold, TLS sequence, AtU3 terminator, AtU3 promoter, Pt4CL1_gRNA, sgRNA scaffold, TLS sequence, AtU3 terminator.
- The right and left T-DNA borders are underlined.

**pLR5479 (**12,697 bp**)**

gtttacccgccaatatatcctgtcaaacactgatagtttaaactgaaggcgggaaacgacaatctgatccaagctcaagctgctctagcattcgccattcaggctgcgcaactgttgggaagggcgatcggtgcgggcctcttcgctattacgccagctggcgaaagggggatgtgctgcaaggcgattaagttgggtaacgccagggttttcccagtcacgacgttgtaaaacgacggccagtgccaagctttcgacgagtcagtaataaacggcgtcaaagtggttgcagccggcacacacgagtcgtgtttatcaactcaaagcacaaatacttttcctcaacctaaaaataaggcaattagccaaaaacaactttgcgtgtaaacaacgctcaatacacgtgtcattttattattagctattgcttcaccgccttagctttctcgtgacctagtcgtcctcgtcttttcttcttcttcttctataaaacaatacccaaagagctcttcttcttcacaattcagatttcaatttctcaaaatcttaaaaactttctctcaattctctctaccgtgatcaaggtaaatttctgtgttccttattctctcaaaatcttcgattttgttttcgttcgatcccaatttcgtatatgttctttggtttagattctgttaatcttagatcgaagacgattttctgggtttgatcgttagatatcatcttaattctcgattagggtttcatagatatcatccgatttgttcaaataatttgagttttgtcgaataattactcttcgatttgtgatttctatctagatctggtgttagtttctagtttgtgcgatcgaatttgtagattaatctgagtttttctgattaacagggtaccgggccccccctcgaggcgcgccaagctatcaaacaagtttgtacaaaaaagcaggctccgaattcgcccttcATGGAGGCCTCTCCGGCTTCAGGTCCTCGCCATCTCATGGACCCCCATATCTTTACCTCCAATTTCAATAACGGCATTGGCAGGCACAAGACATACCTCTGTTATGAGGTCGAGCGGCTCGATAACGGAACCTCAGTGAAGATGGACCAACACAGGGGTTTCCTTCATAACCAAGCGAAAAATCTCCTTTGCGGTTTCTATGGGCGGCACGCGGAGCTGAGATTCTTGGATTTGGTCCCGAGCCTCCAGCTGGATCCCGCGCAAATATACCGCGTCACTTGGTTCATATCATGGAGCCCGTGCTTTAGCTGGGGGTGTGCCGGTGAGGTTCGCGCGTTCCTCCAGGAAAACACCCATGTTCGCCTCCGGATTTTCGCAGCGAGGATTTtCGACTATGATCCACTTTACAAAGAGGCCCTCCAAATGCTTAGAGATGCTGGGGCTCAAGTTTCAATTATGACATACGACGAGTTTAAGCATTGTTGGGACACTTTTGTCGATCATCAGGGTTGTCCCTTCCAGCCGTGGGATGGTCTTGATGAGCATTCGCAAGCTTTGAGTGGCAGGCTTCGCGCGATACTTCAGAACCAGGGTAACtcaggttctgagacccctggcacaagtgagtcagcaacacccgagtccaccatggattacaaggaccacgacggggattacaaggaccacgacattgattacaaggatgatgatgacaagatggctccgaagaagaagaggaaggttggcatccacggggtgccagctgctgacaagaagtactcgatcggcctcgCtattgggactaactctgttggctgggccgtgatcaccgacgagtacaaggtgccctcaaagaagttcaaggtcctgggcaacaccgatcggcattccatcaagaagaatctcattggcgctctcctgttcgacagcggcgagacggctgaggctacgcggctcaagcgcaccgcccgcaggcggtacacgcgcaggaagaatcgcatctgctacctgcaggagattttctccaacgagatggcgaaggttgacgattctttcttccacaggctggaggagtcattcctcgtggaggaggataagaagcacgagcggcatccaatcttcggcaacattgtcgacgaggttgcctaccacgagaagtaccctacgatctaccatctgcggaagaagctcgtggactccacagataaggcggacctccgcctgatctacctcgctctggcccacatgattaagttcaggggccatttcctgatcgagggggatctcaacccggacaatagcgatgttgacaagctgttcatccagctcgtgcagacgtacaaccagctcttcgaggagaaccccattaatgcgtcaggcgtcgacgcgaaggctatcctgtccgctaggctctcgaagtctcggcgcctcgagaacctgatcgcccagctgccgggcgagaagaagaacggcctgttcgggaatctcattgcgctcagcctggggctcacgcccaacttcaagtcgaatttcgatctcgctgaggacgccaagctgcagctctccaaggacacatacgacgatgacctggataacctcctggcccagatcggcgatcagtacgcggacctgttcctcgctgccaagaatctgtcggacgccatcctcctgtctgatattctcagggtgaacaccgagattacgaaggctccgctctcagcctccatgatcaagcgctacgacgagcaccatcaggatctgaccctcctgaaggcgctggtcaggcagcagctccccgagaagtacaaggagatcttcttcgatcagtcgaagaacggctacgctgggtacattgacggcggggcctctcaggaggagttctacaagttcatcaagccgattctggagaagatggacggcacggaggagctgctggtgaagctcaatcgcgaggacctcctgaggaagcagcggacattcgataacggcagcatcccacaccagattcatctcggggagctgcacgctatcctgaggaggcaggaggacttctaccctttcctcaaggataaccgcgagaagatcgagaagattctgactttcaggatcccgtactacgtcggcccactcgctaggggcaactcccgcttcgcttggatgacccgcaagtcagaggagacgatcacgccgtggaacttcgaggaggtggtcgacaagggcgctagcgctcagtcgttcatcgagaggatgacgaatttcgacaagaacctgccaaatgagaaggtgctccctaagcactcgctcctgtacgagtacttcacagtctacaacgagctgactaaggtgaagtatgtgaccgagggcatgaggaagccggctttcctgtctggggagcagaagaaggccatcgtggacctcctgttcaagaccaaccggaaggtcacggttaagcagctcaaggaggactacttcaagaagattgagtgcttcgattcggtcgagatctctggcgttgaggaccgcttcaacgcctccctggggacctaccacgatctcctgaagatcattaaggataaggacttcctggacaacgaggagaatgaggatatcctcgaggacattgtgctgacactcactctgttcgaggaccgggagatgatcgaggagcgcctgaagacttacgcccatctcttcgatgacaaggtcatgaagcagctcaagaggaggaggtacaccggctgggggaggctgagcaggaagctcatcaacggcattcgggacaagcagtccgggaagacgatcctcgacttcctgaagagcgatggcttcgcgaaccgcaatttcatgcagctgattcacgatgacagcctcacattcaaggaggatatccagaaggctcaggtgagcggccagggggactcgctgcacgagcatatcgcgaacctcgctggctcgccagctatcaagaaggggattctgcagaccgtgaaggttgtggacgagctggtgaaggtcatgggcaggcacaagcctgagaacatcgtcattgagatggcccgggagaatcagaccacgcagaagggccagaagaactcacgcgagaggatgaagaggatcgaggagggcattaaggagctggggtcccagatcctcaaggagcacccggtggagaacacgcagctgcagaatgagaagctctacctgtactacctccagaatggccgcgatatgtatgtggaccaggagctggatattaacaggctcagcgattacgacgtcgatcatatcgttccacagtcattcctgaaggatgactccattgacaacaaggtcctcaccaggtcggacaagaaccggggcaagtctgataatgttccttcagaggaggtcgttaagaagatgaagaactactggcgccagctcctgaatgccaagctgatcacgcagcggaagttcgataacctcacaaaggctgagaggggcgggctctctgagctggacaaggcgggcttcatcaagaggcagctggtcgagacacggcagatcactaagcacgttgcgcagattctcgactcacggatgaacactaagtacgatgagaatgacaagctgatccgcgaggtgaaggtcatcaccctgaagtcaaagctcgtctccgacttcaggaaggatttccagttctacaaggttcgggagatcaacaattaccaccatgcccatgacgcgtacctgaacgcggtggtcggcacagctctgatcaagaagtacccaaagctcgagagcgagttcgtgtacggggactacaaggtttacgatgtgaggaagatgatcgccaagtcggagcaggagattggcaaggctaccgccaagtacttcttctactctaacattatgaatttcttcaagacagagatcactctggccaatggcgagatccggaagcgccccctcatcgagacgaacggcgagacgggggagatcgtgtgggacaagggcagggatttcgcgaccgtcaggaaggttctctccatgccacaagtgaatatcgtcaagaagacagaggtccagactggcgggttctctaaggagtcaattctgcctaagcggaacagcgacaagctcatcgcccgcaagaaggactgggatccgaagaagtacggcgggttcgacagccccactgtggcctactcggtcctggttgtggcgaaggttgagaagggcaagtccaagaagctcaagagcgtgaaggagctgctggggatcacgattatggagcgctccagcttcgagaagaacccgatcgatttcctggaggcgaagggctacaaggaggtgaagaaggacctgatcattaagctccccaagtactcactcttcgagctggagaacggcaggaagcggatgctggcttccgctggcgagctgcagaaggggaacgagctggctctgccgtccaagtatgtgaacttcctctacctggcctcccactacgagaagctcaagggcagccccgaggacaacgagcagaagcagctgttcgtcgagcagcacaagcattacctcgacgagatcattgagcagatttccgagttctccaagcgcgtgatcctggccgacgcgaatctggataaggtcctctccgcgtacaacaagcaccgcgacaagccaatcagggagcaggctgagaatatcattcatctcttcaccctgacgaacctcggcgcccctgctgctttcaagtacttcgacacaactatcgatcgcaagaggtacacaagcactaaggaggtcctggacgcgaccctcatccaccagtcgattaccggcctctacgagacgcgcatcgacctgtctcagctcgggggcgacaagcggccagcggcgacgaagaaggcggggcaggcgaagaagaagaagggagacggatcaggcgggtcaacaaacctcagtgacatcatagagaaggaaactggtaagcaactggttattcaagagagcattctgatgctccctgaggaagtcgaggaagttataggaaacaagcctgagagcgatatactcgtgcatacggcgtatgacgagagcacggatgaaaatgtgatgctcttgaccagcgatgcgccagaatataaaccctgggcactggttattcaggactctaacggggaaaataaaatcaagatgctctcaggtggctccccaaagaagaaacgcaaggtttagATTATCAGAGTGGTGGTGGGCCCATAACCCACAGGTCCGCTCTGATgacgtccgatcgttcaaacatttggcaataaagtttcttaagattgaatcctgttgccggtcttgcgatgattatcatataatttctgttgaattacgttaagcatgtaataattaacatgtaatgcatgacgttatttatgagatgggtttttatgattagagtcccgcaattatacatttaatacgcgatagaaaacaaaatatagcgcgcaaactaggataaattatcgcgcgcggtgtcatctatgttactagatcgggaattgatcccccctcgacagcttccggaaagggcgaattcgcaactttgtatacaaaagttgccccatggcgttccctctagataacgcaggatccccaagtggtggctattcgacgagtcagtaataaacggcgtcaaagtggttgcagccggcacacacgagtcgtgtttatcaactcaaagcacaaatacttttcctcaacctaaaaataaggcaattagccaaaaacaactttgcgtgtaaacaacgctcaatacacgtgtcattttattattagctattgcttcaccgccttagctttctcgtgacctagtcgtcctcgtcttttcttcttcttcttctataaaacaatacccaaagagctcttcttcttcacaattcagatttcaatttctcaaaatcttaaaaactttctctcaattctctctaccgtgatcaaggtaaatttctgtgttccttattctctcaaaatcttcgattttgttttcgttcgatcccaatttcgtatatgttctttggtttagattctgttaatcttagatcgaagacgattttctgggtttgatcgttagatatcatcttaattctcgattagggtttcatagatatcatccgatttgttcaaataatttgagttttgtcgaataattactcttcgatttgtgatttctatGtagatctggtgttagtttctagtttgtgcgatcgaatttgtcgattaatctgagtttttctgattaacagAATGGTGACAGGGGGAATGGCAAGCAAGTGGGATCAGAAGGGTATGGACATTGCCTATGAGGAGGCGGCCTTAGGTTACAAAGAGGGTGGTGTTCCTATTGGCGGATGTCTTATCAATAACAAAGACGGAAGTGTTCTCGGTCGTGGTCACAACATGAGATTTCAAAAGGGATCCGCCACACTACATGGTGAGATCTCCACTTTGGAAAACTGTGGGAGATTAGAGGGCAAAGTGTACAAAGATACCACTTTGTATACGACGCTGTCTCCATGCGACATGTGTACAGGTGCCATCATCATGTATGGTATTCCACGCTGTGTTGTCGGTGAGAACGTTAATTTCAAAAGTAAGGGCGAGAAATATTTACAAACTAGAGGTCACGAGGTTGTTGTTGTTGACGATGAGAGGTGTAAAAAGATCATGAAACAATTTATCGATGAAAGACCTCAGGATTGGTTTGAAGATATTGGTGAGgctTCCGccAAGATCGTGGAAGTCAAACACCCACTCGTCAAACACAAGCTGGGACTGATGCGTGAGCAAGATATCAGCACCAAGCGCTTTCGCGAACTCGCTTCCGAAGTGGGTAGCCTGCTGACTTACGAAGCGACCGCCGACCTCGAAACGGAAAAAGTAACTATCGAAGGCTGGAACGGCCCGGTAGAAATCGACCAGATCAAAGGTAAGAAAATTACCGTTGTGCCAATTCTGCGTGCGGGTCTTGGTATGATGGACGGTGTGCTGGAAAACGTTCCGAGCGCGCGCATCAGCGTTGTCGGTATGTACCGTAATGAAGAAACGCTGGAGCCGGTACCGTACTTCCAGAAACTGGTTTCTAACATCGATGAGCGTATGGCGCTGATCGTTGACCCAATGCTGGCAACCGGTGGTTCCGTTATCGCGACCATCGACCTGCTGAAAAAAGCGGGCTGCAGCAGCATCAAAGTTCTGGTGCTGGTAGCTGCGCCAGAAGGTATCGCTGCGCTGGAAAAAGCGCACCCGGACGTCGAACTGTATACCGCATCGATTGATCAGGGACTGAACGAGCACGGATACATTATTCCGGGCCTCGGCGATGCCGGTGACAAAATCTTTGGTACGAAAtaGccgatcgttcaaacatttggcaataaagtttcttaagattgaatcctgttgccggtcttgcgatgattatcatataatttctgttgaattacgttaagcatgtaataattaacatgtaatgcatgacgttatttatgagatgggtttttatgattagagtcccgcaattatacatttaatacgcgatagaaaacaaaatatagcgcgcaaactaggataaattatcgcgcgcggtgtcatctatgttactagatcgggaattgatcccccctcgacagcatgTTTACTTTAAATTTTTCTTATGGCTCAGCCTGTGATGGATAACTGAATCAAACAAATGGCGTCTGGGTTTAAGAAcATCTGTTTTGGCTATGTTGGACGAAACAAGTGAACTTTTAGGATCAACTTCAGTTTATATACGGAGCTTATATCGAGCAATAAGATAAGTGGGCTTTTTATGTAATTTAATGGGCTATCGTCCATATATTCACTAATACCCATGCCCAGTACCCATGTATGCGTTTCATATAAGCTCCTAATTTCTCCCACATCGCTCAAATCTAAACAAATCTTGTTGTATATATAACACTGAGGGAGCACCATTGGTCACAGGTTCCGCGGCGAATGATGTTTTAGAGCTAGAAATAGCAAGTTAAAATAAGGCTAGTCCGTTATCAACTTGAAAAAGTGGCACCGAGTCGGTGCATTATCAGAGTGGTGGTGGGCCCATAACCCACAGGTCCGCTCTGATTCCCTTTCCTTTTTTCTTTTTTTTGCCATAAACTTAAATTTGTATATCGATCATTGTAGATATTGAAAACCTAGAACAAACCAACATCCATGTGAATGTCTTTCATGACTGATTTAGAGATAATTCTTGAATTTTGGAACTAGAATCTATAATGAGCCTaaattaaaacattgtgggacTTTACTTTAAATTTTTCTTATGGCTCAGCCTGTGATGGATAACTGAATCAAACAAATGGCGTCTGGGTTTAAGAAcATCTGTTTTGGCTATGTTGGACGAAACAAGTGAACTTTTAGGATCAACTTCCGTTTATATACGGAGCTTATATCGAGCAATAAGATAAGTGGGCTTTTTATGTAATTTAATGGGCTATCGTCCATATATTCACTAATACCCATGCCCAGTACCCATGTATGCGTTTCATATAAGCTCCTAATTTCTCCCACATCGCTCAAATCTAAACAAATCTTGTTGTATATATAACACTGAGGGAGCACCATTGGTCAAGTGTGGCTCAACAGGTAGAGTTTTAGAGCTAGAAATAGCAAGTTAAAATAAGGCTAGTCCGTTATCAACTTGAAAAAGTGGCACCGAGTCGGTGCATTATCAGAGTGGTGGTGGGCCCATAACCCACAGGTCCGCTCTGATTCCCTTTCCTTTTTTCTTTTTTTTGCCATAAACTTAAATTTGTATATCGATCATTGTAGATATTGAAAACCTAGAACAAACCAACATCCATGTGAATGTCTTTCATGACTGATTTAGAGATAATTCTTGAATTTTGGAACTAGAATCTATAATGAGCCTaaattaaaacattgtgCCAGactagtaagggcAaattcgacccagctttcttgtacaaagtggttcgataattcttaattaactagttctagagcggccgccaccgcggtggagctcgaatttccccgatcgttcaaacatttggcaataaagtttcttaagattgaatcctgttgccggtcttgcgatgattatcatataatttctgttgaattacgttaagcatgtaataattaacatgtaatgcatgacgttatttatgagatgggtttttatgattagagtcccgcaattatacatttaatacgcgatagaaaacaaaatatagcgcgcaaactaggataaattatcgcgcgcggtgtcatctatgttactagatcgggaattcgtaatcatgtcatagctgtttcctgtgtgaaattgttatccgctcacaattccacacaacatacgagccggaagcataaagtgtaaagcctggggtgcctaatgagtgagctaactcacattaattgcgttgcgctcactgcccgctttccagtcgggaaacctgtcgtgccagctgcattaatgaatcggccaacgcgcggggagaggcggtttgcgtattggctagagcagcttgccaacatggtggagcacgacactctcgtctactccaagaatatcaaagatacagtctcagaagaccaaagggctattgagacttttcaacaaagggtaatatcgggaaacctcctcggattccattgcccagctatctgtcacttcatcaaaaggacagtagaaaaggaaggtggcacctacaaatgccatcattgcgataaaggaaaggctatcgttcaagatgcctctgccgacagtggtcccaaagatggacccccacccacgaggagcatcgtggaaaaagaagacgttccaaccacgtcttcaaagcaagtggattgatgtgaacatggtggagcacgacactctcgtctactccaagaatatcaaagatacagtctcagaagaccaaagggctattgagacttttcaacaaagggtaatatcgggaaacctcctcggattccattgcccagctatctgtcacttcatcaaaaggacagtagaaaaggaaggtggcacctacaaatgccatcattgcgataaaggaaaggctatcgttcaagatgcctctgccgacagtggtcccaaagatggacccccacccacgaggagcatcgtggaaaaagaagacgttccaaccacgtcttcaaagcaagtggattgatgtgatatctccactgacgtaagggatgacgcacaatcccactatccttcgcaagacccttcctctatataaggaagttcatttcatttggagaggacacgctgaaatcaccagtctctctctacaaatctatctctctcgagctttcgcagatccggggggcaatgagatatgaaaaagcctgaactcaccgcgacgtctgtcgagaagtttctgatcgaaaagttcgacagcgtctccgacctgatgcagctctcggagggcgaagaatctcgtgctttcagcttcgatgtaggagggcgtggatatgtcctgcgggtaaatagctgcgccgatggtttctacaaagatcgttatgtttatcggcactttgcatcggccgcgctcccgattccggaagtgcttgacattggggagtttagcgagagcctgacctattgcatctcccgccgttcacagggtgtcacgttgcaagacctgcctgaaaccgaactgcccgctgttctacaaccggtcgcggaggctatggatgcgatcgctgcggccgatcttagccagacgagcgggttcggcccattcggaccgcaaggaatcggtcaatacactacatggcgtgatttcatatgcgcgattgctgatccccatgtgtatcactggcaaactgtgatggacgacaccgtcagtgcgtccgtcgcgcaggctctcgatgagctgatgctttgggccgaggactgccccgaagtccggcacctcgtgcacgcggatttcggctccaacaatgtcctgacggacaatggccgcataacagcggtcattgactggagcgaggcgatgttcggggattcccaatacgaggtcgccaacatcttcttctggaggccgtggttggcttgtatggagcagcagacgcgctacttcgagcggaggcatccggagcttgcaggatcgccacgactccgggcgtatatgctccgcattggtcttgaccaactctatcagagcttggttgacggcaatttcgatgatgcagcttgggcgcagggtcgatgcgacgcaatcgtccgatccggagccgggactgtcgggcgtacacaaatcgcccgcagaagcgcggccgtctggaccgatggctgtgtagaagtactcgccgatagtggaaaccgacgccccagcactcgtccgagggcaaagaaatagagtagatgccgaccgggatctgtcgatcgacaagctcgagtttctccataataatgtgtgagtagttcccagataagggaattagggttcctatagggtttcgctcatgtgttgagcatataagaaacccttagtatgtatttgtatttgtaaaatacttctatcaataaaatttctaattcctaaaaccaaaatccagtactaaaatccagatcccccgaattaattcggcgttaattcagtacattaaaaacgtccgcaatgtgttattaagttgtctaagcgtcaatttgtttacaccacaatatatcctgcca
